# Disturbance Regimes Predictably Alter Diversity in an Ecologically Complex Bacterial System

**DOI:** 10.1128/mBio.01372-16

**Published:** 2016-12-20

**Authors:** Sean M. Gibbons, Monika Scholz, Alan L. Hutchison, Aaron R. Dinner, Jack A. Gilbert, Maureen L. Coleman

**Affiliations:** a Graduate Program in Biophysical Sciences, University of Chicago, Chicago, Illinois, USA; b Institute for Genomics and Systems Biology, Argonne National Laboratory, Argonne, Illinois, USA; c Medical Scientist Training Program, University of Chicago, Chicago, Illinois, USA; d Department of Chemistry, University of Chicago, Chicago, Illinois, USA; e James Franck Institute, University of Chicago, Chicago, Illinois, USA; f Department of Ecology and Evolution, University of Chicago, Chicago, Illinois, USA; g Marine Biological Laboratory, Woods Hole, Massachusetts, USA; h Department of Surgery, University of Chicago, Chicago, Illinois, USA; i Department of the Geophysical Sciences, University of Chicago, Chicago, Illinois, USA

## Abstract

Diversity is often associated with the functional stability of ecological communities from microbes to macroorganisms. Understanding how diversity responds to environmental perturbations and the consequences of this relationship for ecosystem function are thus central challenges in microbial ecology. Unimodal diversity-disturbance relationships, in which maximum diversity occurs at intermediate levels of disturbance, have been predicted for ecosystems where life history tradeoffs separate organisms along a disturbance gradient. However, empirical support for such peaked relationships in macrosystems is mixed, and few studies have explored these relationships in microbial systems. Here we use complex microbial microcosm communities to systematically determine diversity-disturbance relationships over a range of disturbance regimes. We observed a reproducible switch between community states, which gave rise to transient diversity maxima when community states were forced to mix. Communities showed reduced compositional stability when diversity was highest. To further explore these dynamics, we formulated a simple model that reveals specific regimes under which diversity maxima are stable. Together, our results show how both unimodal and non-unimodal diversity-disturbance relationships can be observed as a system switches between two distinct microbial community states; this process likely occurs across a wide range of spatially and temporally heterogeneous microbial ecosystems.

## IMPORTANCE

The diversity of microbial communities is linked to the functioning and stability of ecosystems. As humanity continues to impact ecosystems worldwide, and as diet and disease perturb our own commensal microbial communities, the ability to predict how microbial diversity will respond to disturbance is of critical importance. Using microbial microcosm experiments, we find that community diversity responds to different disturbance regimes in a reproducible and predictable way. Maximum diversity occurs when two communities, each suited to different environmental conditions, are mixed due to disturbance. This maximum diversity is transient except under specific regimes. Using a simple mathematical model, we show that transient unimodality is likely a common feature of microbial diversity-disturbance relationships in fluctuating environments.

## INTRODUCTION

Microbial communities are the foundation of all ecosystems on Earth (1). Microbes live in fluctuating environments, and this heterogeneity influences their ecological structure and diversity (2). Similar to what has been found in large-scale ecosystems (3), diversity in microbial systems is often linked to ecological function and stability. For example, recent studies have revealed that higher community evenness is associated with improved functional stability in microcosms containing denitrifying bacteria (4, 5). Similarly, the diversity of natural phytoplankton communities has been associated with increased resource use efficiency and ecological stability (6, 7). In the human gut, microbial diversity appears to be connected with community stability and host health (8, 9). Thus, in order to predict how disturbance might impact ecosystem function, it is important to determine whether there are general rules governing the relationship between microbial community diversity and environmental change.

Disturbances introduce spatiotemporal heterogeneity to an environment and push ecosystems outside their normal range of variability, generally resulting in differential mortality and/or growth of community members. To better understand diversity-disturbance relationships (DDRs) in microbial systems, we can turn to the decades of literature surrounding this topic from traditional ecology (10–14). Despite important differences between microbes and macrobes, such as higher passive dispersal rates (15) and the potential for mixing together of entire microbial ecosystems (16), ecological systems often behave quite similarly across vastly different scales (17–20). In systems ranging from forests to coral reefs to grasslands, maximal diversity has been observed at intermediate frequencies or intensities of disturbance (21–26). The intermediate disturbance hypothesis (IDH) postulates that these peaks in diversity at intermediate levels of disturbance stem from the coexistence of organisms with different life history traits, such as those defined by competition-colonization tradeoffs, along a successional gradient (21, 22, 27, 28). However, alternative non-unimodal DDRs, in which diversity peaks at low or high levels of disturbance, are also frequently detected in nature (29–31).

Here we sought to systematically characterize the relationship between disturbance rate (i.e., the product of disturbance intensity and frequency) and diversity in a microbial ecosystem (32). Prior work using a single-strain system (*Pseudomonas fluorescens*) showed that coexistence of colony morphotypes peaked at intermediate levels of disturbance and productivity (33–35), but it is unclear whether these results translate to more complex communities. Recent laboratory work has shown that multispecies microbial community responses to disturbance depend not only on intensity but also on the frequency of disturbance application (34, 36), suggesting that a simple universal relationship between diversity and disturbance may not exist. We expanded on this prior work and developed an experimental system comprised of a complex bacterial community enriched from Lake Ontario, wherein we could precisely define the dimensions of disturbance (e.g., disturbance type, range, intensity, and frequency) and quantitatively sample community diversity. We imposed disturbance regimes ranging from no applied disturbance to a near-total collapse of ecosystem biomass, and frequency and intensity were independently modulated to assess potential interactions between these factors (34, 37). Further, we employed two qualitatively different disturbances: biomass removal and dilution, which is commonly applied in microcosm studies and is predicted to maintain relative taxon abundances (33–35), and UV radiation, which should induce taxon-specific mortality (38). We predicted that biomass removal/dilution would act as an indiscriminate mortality event with no marked effect on niche-defining environmental properties and therefore would not alter community diversity; we predicted that UV, on the other hand, would give rise to a unimodal DDR due to the coexistence of resistance phenotypes and tradeoffs between resistance and other traits. We show that our results are consistent with a minimal model of resource-coupled species, and we map the landscape of possible DDRs for the model to set our observations in broader context. Together our experimental and modeling results suggest conditions under which a variety of unimodal and non-unimodal DDRs can be observed, not only in microbial systems, but potentially in macroscale ecosystems as well.

## RESULTS

### Empirical results. (i) Community succession occurs in the absence of disturbance.

**FIG 1:**
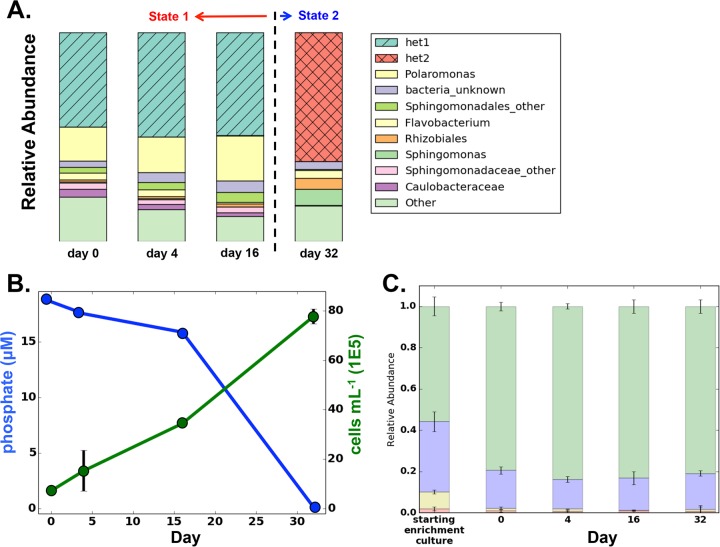
Behavior of the undisturbed control communities over the 32-day experiment. (A) Proportional composition of heterotroph genera across sampling time points. (Only the top 10 most abundant genera are displayed; all other genera are grouped together as “Other.”) Taxonomic annotations represent the most resolved identification based on the Greengenes database. The het1 and het2 OTUs comprise >99% of the sequence reads in their respective genera. (B) The phosphate concentration remained above 15 μM through day 16 and then fell below the detection limit (<1 μM) by day 32 (blue line); the total cell density measured by flow cytometry more than doubled over the same period (green line). (Error bars show standard deviation [SD]; lack of an error bar means the error is smaller than the marker diameter.) (C) Phylum-level community composition of the starting enrichment culture (grown in flask) and undisturbed controls (grown in 96-well plates) through time (green, *Cyanobacteria*; blue, *Proteobacteria*; yellow, *Bacteroidetes*; red, other). Error bars show SD of relative abundances.

Our model system, a freshwater enrichment community, represents a simplified multitrophic ecosystem, with a single dominant cyanobacterium and, on average, dozens of heterotrophic bacteria. We maintained this freshwater enrichment community for months prior to starting our disturbance experiments and observed reproducible growth and community succession (see Materials and Methods and see Table S1 in the supplemental material). Then, this enrichment stock culture was used to inoculate systematic disturbance treatments, as well as undisturbed controls, and we measured responses in community diversity using amplicon sequencing of the 16S rRNA gene. In the undisturbed control communities, the total cell concentration increased 10-fold over the 32-day experiment (Fig. 1). Throughout the course of the experiment, a single cyanobacterial operational taxonomic unit (OTU) closely related to *Synechococcus elongatus* comprised ~80% of sequence reads in the undisturbed controls, and the remaining ~20% of reads represented a diverse, heterotroph community dominated by *Proteobacteria* (Fig. 1; see Text S1 in the supplemental material).

Previous modeling work suggests that DDRs can be obscured when looking across trophic levels (39), so we focused our analysis on the diverse heterotroph community within each sample. In the undisturbed controls, a single OTU dominated the heterotrophic community for the first 16 days, comprising ~50% of the community (class *Alphaproteobacteria*, order *Sphingomonadales*, family *Sphingomonadaceae*, hereafter called het1 [Fig. 1A; Text S1]). Other abundant taxa in the het1-dominated community included *Polaromonas*, *Caulobacteraceae*, and other *Sphingomonadales* OTUs (with taxonomic names representing the most resolved Greengenes annotation available [Fig. 1A]). By day 32, however, community structure changed dramatically, and a second OTU became dominant, comprising ~60% of the heterotrophic community (order *Sphingomonadales*, family *Erythrobacteraceae*, hereafter called het2 [Fig. 1A]). The het2-dominated community was associated with a rise in *Rhizobiales* and *Sphingomonas* OTUs that were not abundant in the het1 state (Fig. 1A). This switch was accompanied by a drop in phosphate concentration between days 16 and 32 from 25 μM to <1 μM (Fig. 1B); nitrate never dropped below 40 μM. We saw the same transition between het1 and het2 during maintenance of the enrichment stock culture, which had been diluted 1:1 with fresh medium each month (Table S1). We were unable to directly observe the transition between het1- and het2-dominated communities in the controls (i.e., a mixed state containing significant levels of both het1 and het2) due to a lack of sufficient temporal resolution.

### (ii) Disturbance promotes a switch between states.

**FIG 2:**
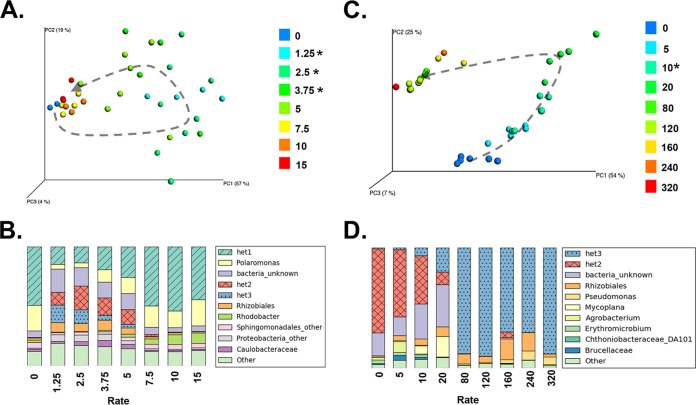
Response of microbial community structure to disturbance rate. (A) Principal coordinate plot (PCoA) showing community structure similarity among biomass removal treatments on day 16, colored by disturbance rate. The gray dashed arrow indicates approximate trajectory of samples in ordination space along the disturbance gradient. Asterisks in panel A indicate disturbance treatments with significantly greater variability among replicates than the control (PERMDISP, *P* < 0.05). (B) Relative abundances of heterotroph genera by biomass removal disturbance rate on day 16. (Only the 10 most abundant genera are shown.) The het1, het2, and het3 OTUs each comprise >99% of the sequence reads that mapped to their respective genera. (C) PCoA showing community structure similarity among UV treatments on day 16 (second experiment), as in panel A. (D) Relative abundances of heterotroph genera across UV disturbance rates on day 16, as in panel B.

Biomass removal disturbances were performed by removing a percentage of the culture volume and replenishing the lost volume with fresh BG11 medium. Hence each biomass removal disturbance also represents a resupply of dissolved inorganic nutrients and dilution of any accumulated organic carbon and other exudates. The highest disturbance rates (rate of 10 or 15% of volume removed per day, where rate is defined as disturbance intensity × frequency), maintained a constant low biomass over time, indicating that cells were doubling at approximately the same rate as they were removed, mimicking a semicontinuous culture. In contrast, biomass removal treatments of lower frequency and/or intensity caused biomass to increase faster than the controls (see Fig. S1 in the supplemental material), suggesting that dilution with fresh medium relieved some growth limitation by a dissolved nutrient or by gas exchange for both the cyanobacterium and its coexisting heterotrophs. After being subjected to each disturbance regime for 4 days, community structures remained similar across all disturbance treatments and unchanged from those of the undisturbed controls (multiresponse permutation procedure [MRPP], *P* > 0.1) (see Fig. S2A in the supplemental material). By day 16, however, community structure varied significantly depending on disturbance rate (MRPP, *P* < 0.01) (Fig. 2A and B; Fig. S2A). Surprisingly, community structure was most similar between the undisturbed controls and the highest-disturbance-rate treatments (rate of 7.5, 10, or 15% volume replacement day^−1^); the low to intermediate disturbance rates, where growth outpaced undisturbed controls, induced strong changes in structure and high variation among replicates (Fig. 2A and B).

Phosphate concentration correlated with community structure: when phosphate was greater than 15 µM, the community was in a het1-dominated state, and when phosphate was below detection, the community was het2 dominated (see Fig. S3 in the supplemental material). Intermediate levels of phosphate coincided with mixtures of the het1- and het2-dominated states. Despite this correlation, phosphate may not be the true driver of the community switch but instead may reflect other concomitant changes in environmental conditions. For example, more rapid growth of the cyanobacterium in the low- to intermediate-disturbance-rate treatments (Fig. S1), due to resupply of a growth-limiting resource (probably not phosphate, since the P concentration was still >15 µM in the undisturbed controls on day 16), may have altered organic carbon pools via exudation and selected for a distinct heterotroph community. Five of the top 10 heterotroph taxa belong to the order *Sphingomonadales* (*Alphaproteobacteria*), including het1 and het2 (Text S1); members of this group are known to degrade a variety of simple sugars and polysaccharides, with a high degree of strain specificity (40), and have been found in close association with algae (41). Besides phosphate and organic carbon, other nutrients, oxygen, or waste products could also be responsible for the transition.

On day 32, the biomass removal disturbance regimes no longer preserved a range of phosphate concentrations; instead, the system fell into high- or low-phosphate extremes (see Fig. S4 in the supplemental material), presumably associated with other environmental differences as well. These two extremes were associated with distinct community structures, dominated by het1 in the highest-rate treatments and het2 in all other treatments (Fig. S2A). Thus, as standing biomass increased in all but the highest-removal-rate treatments (rate of 10 or 15% day^−1^), the rate of phosphate consumption likely increased, phosphate was drawn down, other conditions may have simultaneously changed (such as organic substrate availability), and the community switched to a het2-dominated state.

### (iii) UV disturbances also modulate switching between community states.

We chose UV radiation as a qualitatively different disturbance type from biomass removal, postulating that UV would induce taxon-specific mortality. In addition to direct killing of UV-sensitive cells, UV could change community structure through induction of lysogens and altered nutrient dynamics in the wake of cell death and could also induce mutations in surviving taxa. Initially we subjected communities to various rates of UV exposure, up to 15 min per day: yet even at this maximum disturbance rate, biomass continued to increase, albeit more slowly than in the undisturbed controls (see Fig. S5 in the supplemental material). Therefore, we performed a second experiment with higher rates of UV exposure, in which we spanned a sufficiently wide range of disturbance rates to cause biomass collapse (see Fig. S6 in the supplemental material). For disturbance rates above 5 min of UV exposure day^−1^, UV disturbances altered community composition as early as day 4 (MRPP, *P* < 0.002 [Fig. S2B]). After day 4, all UV disturbance regimes significantly altered community composition (MRPP, *P* < 0.01 [Fig. S2B and C]). Unlike biomass removal, higher UV disturbance rates caused communities to become less similar to those of the undisturbed controls (Fig. 2C and D). UV disturbances were not associated with the het1/het2 transition (Fig. S2B and C). Rather, another OTU, presumably UV tolerant, became dominant at high UV rates (also in the order *Sphingomonadales*, genus *Blastomonas*; hereafter called het3), comprising ~80% of the heterotroph community (Fig. 2D). The het3-dominated state was also associated with a rise in *Rhizobiales* and *Pseudomonas* OTUs (Fig. 2D).

### (iv) Community variance peaks at intermediate disturbances.

For both biomass removal and UV, low to intermediate disturbance rates produced higher variance in community structure among replicates relative to undisturbed controls. After 16 days of biomass removal disturbance treatments, variance was significantly higher for disturbance rates from 1.25 to 5% day^−1^ than for the controls and high-rate disturbances (permutational analysis of multivariate dispersions [PERMDISP], *P* < 0.05 [Fig. 2A]). Under UV disturbance regimes, variance among replicates was significantly higher for the intermediate disturbance rate at 10 min of exposure day^−1^, when compared to controls and high-disturbance-rate treatments (PERMDISP, *P* < 0.04 [Fig. 2C]).

### (v) Empirical DDRs.

**FIG 3:**
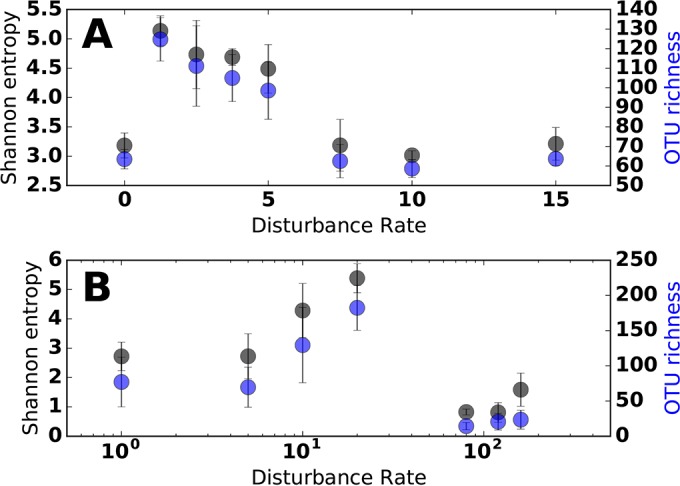
Relationship between microbial community alpha diversity (black, Shannon entropy; blue, OTU richness) and disturbance rate. (A) Diversity versus disturbance rate for biomass removal treatments on day 16. (B) Diversity versus disturbance rate for UV treatments on day 16. Error bars show SD.

Across a range of biomass removal regimes, diversity was significantly higher at intermediate-disturbance rates (1.5, 2.5, 3.75, and 5; 2-tailed *t* test, *P* < 0.05) on day 16 (Fig. 3A; see Fig. S7 in the supplemental material), when het1 and het2 community assemblages coexisted (Fig. 2B). This coexistence was likely associated with short-term temporal heterogeneity in phosphate (and other resource) concentrations (Fig. S3 and S4) fluctuating with the removal of biomass and resupply of fresh medium with each disturbance event. These fluctuations would be less pronounced at early time points when resource availability was high and biomass low and conversely at later time points when high biomass would rapidly consume resources. These results underscore how biological feedbacks can alter the relationship between disturbance and environmental parameters over time, which in turn change the types of DDRs that are observed.

In the second UV experiment, we subjected our microcosms to a wide range of disturbances, which revealed a drop in diversity at the highest disturbance rates (Fig. 3B; Fig. S7). Intermediate rates of UV exposure (10 and 20 min day^−1^) in the second experiment resulted in significantly greater diversity than controls and high-rate disturbances (two-tailed *t* test, *P* < 0.05 [Fig. 3B]), which is likely due to the coexistence of UV-tolerant and UV-sensitive communities (Fig. 2D).

In our system, we found similar DDR patterns using different alpha diversity metrics, including Shannon entropy and OTU richness (Fig. 3). Due to the compositional nature of 16S rRNA amplicon data (i.e., relative abundances), alpha diversity metrics for microbial community richness and evenness are often highly correlated. Thus, detection of DDR patterns is likely to be robust to the type of alpha diversity metric used, which is not necessarily the case for macroecological systems (42).

Our experimental approach was limited in that we could only follow batch microcosm communities for a few generations before growth stopped. Moreover, our sampling resolution was constrained to just a few time points, making it challenging to reconstruct dynamics on shorter time scales. To explore longer-term DDR dynamics, with higher temporal resolution than we could observe empirically, we formulated a simple mathematical model.

### Modeling results: Lotka-Volterra model.

**FIG 4:**
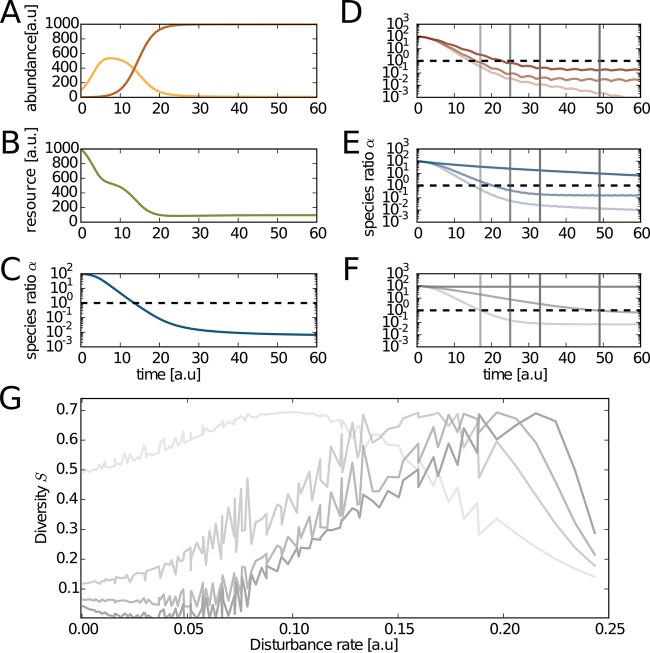
A simplified two-state competitive Lotka-Volterra model for exploring disturbance-diversity relationships. (A) Dynamics of het1 (red) and het2 (orange) for the undisturbed condition. (B) Dynamics of the resource subject to competition in the undisturbed condition. (C) Dynamics of the species ratio α in the undisturbed condition. (D to F) Dynamics of the species ratio α with increasing frequency of disturbance per panel. Increasing color saturation indicates increasing intensity of disturbance. The dashed horizontal line shows the diversity maximum, where the species ratio alpha is equal to 1. (G) DDR for the model at several time points. These time points can be seen as the vertical lines of matching saturation in panels D, E, and F. Increasing saturation of the gray lines indicates later time points. The jagged structure within each DDR reflects coupling between the oscillations of the Lotka-Volterra model and the frequency of disturbance; we expect these effects are exaggerated by the simple structure of the model.

Having observed unimodal DDRs empirically (Fig. 3) on day 16, but not at other sampling points, we wanted to better understand the dynamics that give rise to these patterns and explore conditions that might lead to non-unimodal DDRs. To this end, we developed a basic Lotka-Volterra (LV) consumer-resource model that represents the dynamics of the dominant heterotroph species from the biomass removal experiment, with a resource-dependent carrying capacity to mimic the apparent phosphate dependence of het1 (Text S1). We explicitly introduced dynamic resource levels, where het1 and het2 both consumed a common resource as they grew and released the resource when they died. For simplicity, we assumed equal growth rates and competition parameters, though our results were robust to a range of parameter values (data not shown). Our model recapitulated the switch we observed experimentally in the undisturbed controls (Fig. 1 and Fig. 4A and B). Our model assumes that the switch in dominance between het1 and het2, along with accompanying changes in other taxa, is due to competition for a shared resource that is modified by disturbance events: if, for example, the dominant taxa are specialized for distinct resources whose availability varies through time, then our simple model would not necessarily apply.

To model the biomass removal disturbance, we periodically scaled all variables (het1, het2, and the resource) to a given percentage of their abundance (disturbance intensity) and added back the same percentage of the initial resource level. We assumed a minimum abundance for each species, which allows the inferior competitor to persist at low frequency and recover when conditions become favorable for growth; notably, this precludes competitive exclusion and extinction events. This assumption seems reasonable, as “seed banks” are common features of microbial ecosystems (15, 43, 44).

**FIG 5:**
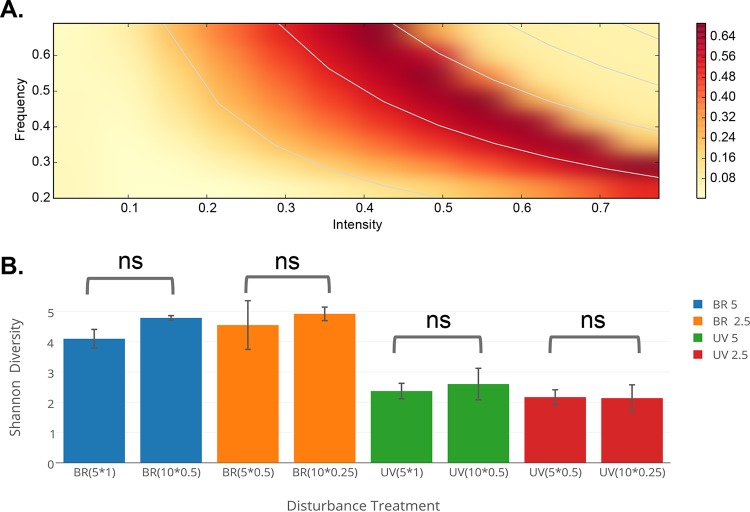
Effect of disturbance frequency and intensity on Shannon diversity for both the model and experiments. (A) Shannon entropy heat map over disturbance intensity and frequency axes for the Lotka-Volterra model. White lines show hyperbolic disturbance rate isoclines. (B) Shannon entropy in disturbance experiments. Shown are cases where multiple combinations of disturbance frequency and intensity gave equivalent disturbance rates. The *x-*axis labels designate the disturbance type (biomass removal or UV), the disturbance intensity (5 or 10 [representing the percentage of volume or minutes of exposure, respectively]), and the frequency (1, 0.5, or 0.25 day^−1^). Error bars show standard deviation. ns, not significant.

We defined the species ratio between het1 and het2, each representing an alternate ecological state, as α. We used the Shannon entropy (*S*) to quantify diversity. *S* is maximal when both species are present at equal proportion. In this model, we defined the two states as high α (α ≫ 1) and low α (α ≪ 1): by switching between states, the identity of the dominant competitor is changed. In the control condition, α decayed to a value determined by the het1 abundance minimum (Fig. 4C). The decay rate and the steady-state α value were both modulated by disturbance treatments (Fig. 4D and F). For low levels of disturbance, the het2 state dominated, as in the control condition (Fig. 4D and F). For intermediate disturbance rates, a nonequilibrium state persisted where het1 and het2 were evenly matched in their abundance. For high levels of disturbance, het1 maintained its dominance over het2 (Fig. 4D and F). The DDR pattern changed shape with time, while maintaining unimodality (Fig. 4G). This transience was due to the feedback between biomass and resource availability, similar to the feedback we propose to explain our experimental results. This effect could impair the detection of unimodal DDRs due to insufficient sampling of the DDR landscape. In contrast to the predictions of another recent DDR model in which the frequency and intensity of the disturbance were coupled in a nonmultiplicative fashion (37), we saw no evidence for transient or persistent U-shaped DDRs (Fig. 5B). The simple model that we present here is sufficient to capture our observations and shows that a disturbance in a common resource can lead to unimodal DDRs.

We initially explored higher-dimensional models (e.g., including the cyanobacterium and other heterotroph community members), but given the low resolution of our empirical data, it was difficult to directly compare the model and experiments. Therefore, we thought it prudent to use the highly simplified, phenomenological model described above, which relies on very few parameters and assumptions. Nevertheless, development of more realistic models should be a priority for future work. Further discussion of the model, including equations and parameter values, can be found in Text S1.

## DISCUSSION

We developed a tractable yet realistic experimental model system for assessing diversity-disturbance relationships in microbial systems. Microcosm communities underwent a reproducible succession between distinct ecological states, each characterized by a dominant species (i.e., het1, het2, and het3). We observed different apparent relationships between diversity and disturbance rate over the course of the experiment, which we propose to be due to the combined effects of biologically induced environmental change (i.e., limiting resource drawdown) and our imposed disturbance regimes. Alternatively, it is possible that community succession could be decoupled from environmental conditions. To test this hypothesis, one would need an experimental system in which environmental conditions could be manipulated separately from the community that normally creates them—for example, with a flowthrough system.

Initially, we did not expect to find changes in community diversity in the biomass removal/dilution treatments, because indiscriminate mortality events alone are not expected to alter relative taxonomic abundances (45). Accordingly, the similarity in community structures between undisturbed controls and the highest-rate biomass removal treatments on day 16 (Fig. 2A and B) suggests that density-independent mortality alone has no detectable effect on community diversity. However, we did observe changes in diversity in other biomass removal treatments, which we suggest were mediated by changes in resource availability. Complementing our results, prior work in *P. fluorescens* microcosms found that biomass removal disturbances modified oxygen concentrations, which in turn altered community composition (46). In our work, we propose that the indirect effect of biomass removal on the abiotic environment shifted over time due to a biological feedback on resource availability, leading to a transient unimodal pattern. Similar dynamics also occurred in our simplified model, but we showed that we could obtain persistent coexistence if the environmental disturbance remained within a small range of rates. If our explanation is correct, such temporal dynamics may partly explain the variety of DDR patterns observed in nature and may be a common feature of both microbial and macrobial systems in fluctuating environments (30).

Recent work has implied that disturbance generally reduces variability in microbial community composition (47, 48), somewhat contradicting our findings (Fig. 2A and C). We found that community structure was more variable among replicates in the high-diversity intermediate-disturbance treatments, which may be symptomatic of a transition toward more stochastic community assembly (49). The lack of community convergence to an “intermediate state” of maximum diversity suggests that DDR peaks are transient, where there are no clear winners or losers. We speculate that peak community variance centered at the apex of a unimodal DDR curve might be a general phenomenon and that this would be an interesting subject for future investigation. Indeed, these results mirror prior work in a grassland ecosystem, where species compositional stability was lowest in high-diversity plots, while functional stability was greatest in those same plots (3).

Our very limited model illustrated how DDR structures can shift over different time scales in the presence of consumer-resource feedbacks (Fig. 4). Diversity responded uniformly to the product of frequency and intensity in the model, which matched what we saw in our more complex enrichment communities (Fig. 5). This multiplicative interaction between intensity and frequency was also observed for a special case of a recent vegetation model (i.e., when age to maturity and dispersal capability were similar across competitors) and is consistent with results from experiments involving coexistence of colony morphotypes across a range of disturbance intensities and frequencies (34). We saw no evidence for nonmultiplicative behaviors in our experiments, e.g., U-shaped DDRs (37). Our model was constructed with independent contributions from frequency and intensity, which is consistent with experimental results (Fig. 5B). However, it is possible that different experimental conditions or a higher-dimensional model might identify these types of complex relationships.

Regardless of whether the IDH is valid, our work highlights the importance of understanding the mechanisms underlying disturbance-induced changes in diversity. In our case, we have framed unimodal DDRs as nonequilibrium mixtures of incompatible ecological states (defined by fitness tradeoffs along an environmental gradient) maintained by disturbance-induced environmental heterogeneity. We suggest that temporally stable DDRs (unimodal or otherwise) are rare due to the ubiquity of ecological and environmental feedbacks (50), which can dampen disturbance-induced environmental heterogeneity. Over longer time scales, evolutionary adaptation can alter the relationships between species traits and the environment, which could in turn alter DDR structure in persistently disturbed ecosystems. Moving forward, it will be important to replicate our results in other controlled systems with higher temporal resolution to assess their generality, investigate how ecosystem function and stability are affected by a community’s position along a DDR curve, and explore the intersection between disturbance-based coexistence and evolution (e.g., the potential for horizontal gene transfer, competition/cooperate tradeoffs, etc.). From the influence of antibiotics on pathogen susceptibility in the gut (51) to eutrophication in freshwater ecosystems (52), more reliable models for how microbial diversity responds to disturbance will inform our ability to predict the ecological stability of microbial systems in the presence of perturbations.

## MATERIALS AND METHODS

### Microcosm community and growth conditions.

Surface water was collected from Lake Ontario in August 2012, prefiltered using a Whatman GF/A glass fiber filter (nominal pore size of 1.6 µm), and used to inoculate 100 ml of BG11 medium (53). BG11 medium contains no organic carbon except for low-concentration chelators, so heterotrophic growth was sustained by primary production from coexisting cyanobacteria. The enrichment was cultivated for 9 months in the presence of cycloheximide (5 μg ml^−1^) to eliminate eukaryotic organisms; after 9 months, cycloheximide was omitted. The enrichment was grown without shaking in a glass bottle at 22°C in an incubator (Percival Scientific) set to a 12-h day-night cycle (maximum light intensity of ~30 microeinsteins m^−2^ s^−1^) and was diluted with 50% fresh medium every ~30 days. Over this 30-day period, there was a reproducible growth pattern: cyanobacterial biomass increased after the addition of fresh medium for 2 to 3 weeks, at which point chlorophyll fluorescence plateaued; the optical density at 600 nm (OD_600_) continued to increase, reflecting a higher relative abundance of heterotrophic cells. The enrichment was maintained for 16 months prior to the first experiment. Taxonomic composition for the enrichment community is described in Table S1, sampled at two time points (i.e., high- and low-phosphate time points).

Disturbance experiments were carried out in 96-well transparent flat-bottom microtiter plates. Two weeks before the start of each experiment, the enrichment was diluted 50% with fresh BG11 medium and grown for 1 week in the bottle with stirring, and then 96-well plates were inoculated with 200 μl of the enrichment culture per well and allowed to acclimate in the plate for 1 week prior to the initiation of disturbance treatments at time zero (*T*0). Plates were grown under the same light and temperature conditions described above.

### Experimental design.

We employed a full-factorial experimental design, modulating disturbance frequency and intensity. Our first disturbance type, biomass removal, encompasses both nonspecific mortality across the entire community and resupply of nutrients via dilution with fresh medium. Biomass removal disturbance was applied by removing 10, 20, or 30 μl (5, 10, or 15%) of volume from each well (after mixing the contents of the well by pipetting), and this volume was replaced with an equivalent volume of fresh (sterile) medium. Disturbance was applied at three different frequencies (every day, every 2 days, and every 4 days [i.e., 1 day^−1^, 0.5 day^−1^, and 0.25 day^−1^]). A qualitatively different disturbance type, UV radiation, was applied using a UV sterilization lamp (G15T8 bulb; emission peak at 254 nm, 5 W) installed in a laminar flow hood (AirClean Systems). The intensity of disturbance was modulated by exposing wells to UV for different durations (5, 10, or 15 min); hence, intensity in minutes is proportional to the integrated number of photons received by a community per disturbance event. As with biomass removal, UV was imposed at three frequencies (1 day^−1^, 0.5 day^−1^, and 0.25 day^−1^). Untreated wells were shielded from UV with aluminum foil. For both biomass removal and UV experiments, disturbance treatments were continued for 32 days. Disturbance rate was calculated as intensity × frequency, in units of percentage of volume replaced per day or minutes of UV exposure per day. In some cases, the same disturbance rate represents two different regimes (e.g., the rate 2.5% day^−1^ corresponds to both 5% × 0.5 day^−1^ and 10% × 0.25 day^−1^). In these cases, we found no significant difference between community diversity in the two regimes (Fig. 5B), so results are shown as the average of both regimes for a given rate (Fig. 2 and 3).

Each disturbance regime was replicated in 4 to 5 wells. Disturbance frequency treatments were replicated in three spatially separated blocks so that most replicates were not adjacent within a plate. Disturbance intensities were organized onto separate plates, whose positions and orientations in the incubator were rotated every day to further prevent location effects. Control wells (undisturbed) were included in all plates. Outer wells contained water only (replenished as needed), and plates were wrapped with Parafilm to prevent evaporation. The outermost wells that were inoculated (adjacent to the water-filled wells) showed more rapid cyanobacterial growth, presumably due to higher light availability. Therefore, replicates from outer wells were not included in the analyses.

During the initial experiment, even in the most severe UV treatment (15 min of exposure every day), only moderate mortality was observed. To span higher severities of UV disturbance, a second experiment was performed. The design and setup were identical to those in the first experiment, except that the outer wells were filled with enrichment culture (rather than water), to prevent differential light availability. The same three disturbance frequencies were used, but with longer durations of UV exposure (20, 40, and 60 min). For the two higher intensities of disturbance (40 and 60 min), plates were also located closer to the UV bulbs (equivalent to an 8-fold increase in UV intensity, compared to other treatments). Disturbance rates for the second UV experiment were calculated as intensity (minutes) × frequency (day^−1^) × intensity multiplier (8 for the 40- and 60-min treatments and 1 for the 20-min treatment), giving rates ranging from 5 min day^−1^ (20 min × 0.25 day^−1^) to 480 min day^−1^ (60 min × 1 day^−1^ × 8). This experiment was carried out for 16 days. The initial OD_600_ was higher at the start of the second experiment (~0.5 compared to ~0.2 in the initial experiment), which may have accelerated the state switch observed in the undisturbed controls to the het2-dominated state.

Samples were collected for sequencing by thoroughly mixing the contents of each well and transferring them to a collection plate. Sacrificed wells on the experimental plate were replenished with deionized water to prevent evaporation from adjacent wells. Samples were harvested by centrifugation at 4,700 × *g* for 15 min at room temperature. Supernatants were frozen at −20°C for nutrient analyses, and cell pellets were frozen at −80°C until DNA extraction.

### Measuring growth and cell concentration.

During the course of the experiments, absorbance (600 nm) and fluorescence (488-nm excitation/670-nm emission) measurements were taken daily, before and after disturbance, using a Tecan Infinite M200pro plate reader (Salzburg, Austria). All plates were shaken for 60 s in the plate reader prior to measurement. A subset of samples was processed for flow cytometry. For each cytometry sample, 10 μl of culture was added to 985 μl of sterile BG11 medium and 5 μl of 25% glutaraldehyde. These samples were vortexed, stored in the dark for 30 min, and then flash-frozen in liquid nitrogen and stored at −80°C prior to analysis. Samples were analyzed on an Attune acoustic focusing cytometer (Life Technologies, Inc.) equipped with violet (405-nm) and blue (488-nm) lasers. Samples were stained with a 1× concentration of SYBR Gold (Life Technologies, Inc.). Cyanobacterial cells were identified by chlorophyll fluorescence, while heterotrophic cells were counted as particles lacking chlorophyll fluorescence that gave a SYBR Gold fluorescence signal.

### Bacterial DNA extraction, amplification, and sequencing.

To assess microbial diversity, we targeted the V4 region of the 16S rRNA gene. Bacterial DNA was extracted in 96-well collection plates using the Ultra-Clean water DNA isolation kit (Mo Bio Laboratories) according to the manufacturer’s instructions. The V4 region of the 16S rRNA gene was amplified using Earth Microbiome Project protocols (http://www.earthmicrobiome.org/emp-standard-protocols/). EMP primers 515F (5′-GTGCCAGCMGCCGCGGTAA-3′) and 806R (5′-GGACTACHVGGGTWTCTAAT-3′) were used for PCR amplification (54). Sequencing was performed on the Illumina MiSeq platform at Argonne National Laboratory (Argonne, IL).

### Diversity analysis.

QIIME (Quantitative Insights into Microbial Ecology, v.1.7.0; http://www.qiime.org) was used to filter reads and cluster operational taxonomic units (OTUs) as described previously (55, 56). Briefly, we used the open reference OTU picking script (pick_open_reference_otus.py) (56), whereby sequences were first clustered with the Greengenes (May 2013) reference database (57); OTUs that did not cluster with known taxa (at 97% identity) in the database were then clustered *de novo*. Singleton sequences were removed prior to downstream analyses. Representative sequences for each remaining OTU were aligned using PyNAST, with a minimum alignment overlap of 75 bp (58), and a phylogenetic tree was estimated using FastTree v2.0 (59). Taxonomic assignments were made using the RDP classifier (60). We computed alpha diversity using the alpha_diversity.py script in QIIME, normalizing sequencing depth across samples. Significant differences in alpha diversity were assessed using a Student’s *t* test (two-tailed, assuming unequal variances). We used QIIME’s beta_diversity_through_plots.py script to compute beta diversity distances between samples and to construct principal coordinate plots using the weighted UniFrac distance metric (61), which accounts for both the phylogenetic composition and the relative abundance of taxa. The composition of the heterotroph community was reproducibly characterized at a sampling depth of 430 sequences per sample (see Fig. S8 in the supplemental material). We tested for significant beta diversity clustering using the nonparametric multiresponse permutation procedure (MRPP), which determines significant differences in sample groupings in multivariate space. Significant differences in intrareplicate variance were assessed with permutational analysis of multivariate dispersions (PERMDISP). Taxonomic summaries were generated using the summarize_taxa_through_plots.py script. Plotting was carried out using the Matplotlib graphics library in Python (62).

### Phosphate and nitrate quantification.

Colorimetric nitrate and phosphate assays were performed as described previously (63, 64).

### Sequence data and metadata.

Raw sequence data and metadata can be publicly accessed on FigShare (http://dx.doi.org/10.6084/m9.figshare.1007711).

## SUPPLEMENTAL MATERIAL

Text S1  Supplemental results from microcosm experiments and development of the mathematical model. Download Text S1, DOCX file, 1.1 MB

Figure S1  Growth curves for biomass removal treatments (OD_600_ and fluorescence at 488/670). Disturbances start at day 1 and extend to day 32. Intensity treatments (5, 10, and 15%) are plotted separately, for clarity. Colors indicate disturbance frequency (red, 1 day^−1^; green, 0.5 day^−1^; yellow, 0.25 day^−1^; blue, undisturbed). Disturbance rates are annotated to the right of each curve. Undisturbed controls are plotted in all panels for comparison. The colored area surrounding lines indicates the SD of spectrophotometric measurements across replicates. Vertical dashed lines show sampling day 16. Download Figure S1, PDF file, 0.5 MB

Figure S2  Relative abundances of heterotroph genera for all biomass removal (A) and UV (B) rates on days 4, 16, and 32 and for the second UV experiment (C). The het1, het2, and het3 OTUs comprise >99% of the sequence reads for their respective genera. Download Figure S2, PDF file, 0.2 MB

Figure S3  Relative abundances of het1 (black) and het2 (red) across a range of phosphate concentrations. All biomass removal treatment data are included across all time points. Download Figure S3, PDF file, 0.05 MB

Figure S4  Relationship between phosphate, biomass, and disturbance treatment across time. (A) Phosphate concentration plotted against fluorescence intensity, colored by disturbance rate (biomass removal treatments). (B) Same plot as in panel A, but points are colored by time point. (C) Phosphate concentration plotted against fluorescence intensity, colored by disturbance rate (UV treatments). (D) Same plot as in panel C, but points are colored by time point. Download Figure S4, PDF file, 0.2 MB

Figure S5  Growth curves for first UV experiment (OD_600_ and fluorescence at 488/670). Disturbances start at day 1 and extend to day 32. Intensity treatments (5, 10, and 15 min) are plotted separately for clarity. Colors indicate disturbance frequency (red, 1 day^−1^; green, 0.5 day^−1^; yellow, 0.25 day^−1^; blue, undisturbed). Disturbance rates are annotated to the right of each curve. The colored area around lines indicates the SD of spectrophotometric measurements across replicates. Vertical dashed lines show sampling day 16. Download Figure S5, PDF file, 0.5 MB

Figure S6  Growth curves for UV treatments from the second experiment (OD_600_ and fluorescence at 488/670). Disturbances start at day 1 and extend to day 16. Intensity treatments (20, 40, and 60 min) are plotted separately, for clarity. Colors indicate disturbance frequency (red, 1 day^−1^; green, 0.5 day^−1^; yellow, 0.25 day^−1^; blue, undisturbed). Disturbance rates are annotated to the right of each curve. The designations 1× and 8× refer to UV intensity. (Intensity was modulated by changing the proximity of the plates to the UV bulb; radiation intensity decreases as the inverse square of the distance from the source.) The colored area around lines indicates the SD of spectrophotometric measurements across replicates. Vertical dashed lines show sampling day 16. Download Figure S6, PDF file, 0.5 MB

Figure S7  Empirically sampled DDRs for biomass removal and UV treatments, along frequency and intensity axes. Panels A and B show Shannon diversity heat maps for biomass removal and UV exposure treatments, respectively, for day 16 (heterotroph community). Diagonal lines indicate unfeasible combinations. (Without disturbance, there can be no variability in disturbance intensity or frequency.) In panel B, “8×” following the highest intensity treatments refers to a higher applied UV intensity (i.e., the plates were brought closer to the lamp, equivalent to an 8-fold increase in UV flux). Download Figure S7, PDF file, 0.1 MB

Figure S8  (A) Jackknifed beta diversity analysis (based on weighted UniFrac distances) colored by time point. Translucent bubbles around points indicate the uncertainty (SD) in the principal coordinates of a sample after multiple resampling (with replacement) at 430 sequences (*n* = 10). Overall, beta diversity patterns are robust to random resampling at 430 sequences per sample, indicating that 430 sequences are enough to accurately describe beta diversity patterns. (B) Jackknifed Shannon diversity analysis for every sample in the data set. Blue bars show mean Shannon diversity, and black lines show the uncertainty (SD) in Shannon diversity after multiple random subsamplings to a depth of 430 sequences per sample (*n* = 10). Download Figure S8, PDF file, 0.6 MB

Table S1  Taxonomic composition of the enrichment stock culture used to initiate disturbance experiments, summarized at the genus level. Relative abundances of 15 dominant heterotrophic genera are shown at two different collection dates, along with the most resolved Greengenes annotation available. The phosphate concentration was high when the sample was collected in August (>10 μM) and low when the sample was collected in November (<1 μM). The genera containing het1, het2, and het3 OTUs are highlighted in bold; the het1, het2, and het3 OTUs each accounted for >99% of the reads in their respective genera. Table S1, PDF file, 0.1 MB
