# Supplementary material for: Disturbance Regimes Predictably Alter Diversity in an Ecologically Complex Bacterial System
Source: mBio. 2016 Dec 20;7(6):e01372-16. doi: 10.1128/mBio.01372-16 (PMC5181773; doi:10.1128/mBio.01372-16)
Supplement: Text S1 — Supplemental results from microcosm experiments and development of the mathematical model. Download [file mbo006163113s1.docx]

**Text S1**

**Supplementary Results**

*Summary of the amplicon dataset*

There were 16,783 non-cyanobacterial OTUs identified across the entire data set, but the vast majority of these taxa were only detected once or twice. After removing all cyanobacterial reads and normalizing the sequencing effort to 430 sequences per sample, OTU richness varied between 9 and 233 (mean = 71) OTUs per sample. To test whether patterns in alpha and beta diversity were robust to subsampling to just 430 sequences, we repeated the subsampling 10 times. Richness and beta diversity estimates were quite stable across multiple rarefactions (Fig. S8; sample IDs are provided in the metadata mapping file, available on FigShare along with all our raw and summarized data: <http://dx.doi.org/10.6084/m9.figshare.1007711>).

*Dynamics of cyanobacteria across disturbance regimes*

Across all treatment groups, as well as the starting enrichment cultures, the mean relative abundance of cyanobacteria was 82% (SD = 18%) based on amplicon sequencing. Cyanobacterial abundances remained above ~70% across the entire data set and never declined below 50%, except in the highest-UV treatments (second UV experiment) where cyanobacterial abundance dropped below 10%. This crash in the proportion of cyanobacteria corresponded with a drop in total biomass in the high-UV treatment (Fig. S6). This disturbance-induced reduction in cyanobacteria as a proportion of the community was not observed in the biomass removal treatments; instead, the relative abundance of cyanobacteria remained high even when total biomass was reduced in the highest biomass removal rate treatments (Fig. S1).

*Taxonomy and metabolic potential of the dominant heterotrophs*

A BLAST search of 16S rRNA amplicons from the dominant heterotrophs showed that het1, het2, and het3 are most similar to *Sphingopyxis wooponensis* (99% identity over length of the amplicon), *Porphyrobacter colymbi*, (100% identity), and *Blastomonas natatoria* (100% identity), respectively. Het2 and het1 share 90.2% sequence identity, while het3 shares 93.4% sequence identity to het1 and 92.7% sequence identity to het2. All three taxa belong to the *Alphaproteobacteria* phylum and the order *Sphingomonadales*, whose members are aerobic, obligate chemoheterotrophs (1). Both het1 and het3 belong to the *Sphingomonadaceae* family, while het2 belongs to the *Erythrobacteraceae*. Some taxa within both the *Sphingomonadaceae* and *Erythrobacteraceae* synthesize bacteriochlorophyll and are capable of photoheterotrophy, though this trait is not universal in either family. In particular, the genera *Porphyrobacter* (2, 3) and *Blastomonas* (4), are known to contain aerobic anoxygenic phototrophs, suggesting that het2 and het3 may also have this capability.

Aerobic anoxygenic phototrophs lack the Calvin cycle and other carbon fixation pathways (apart from anaplerotic reactions), hence they rely on organic carbon for growth (5). The ability to harvest light energy enables the cells to use photophosphorylation for ATP production, and thereby reduce their respiration of organic carbon. This photoheterotrophic metabolism is thought to provide a fitness advantage under carbon-starvation conditions (6). In our experiments, the rise in dominance of het2 and het3 was associated with a drop in phosphate, which may also correlate with a decrease in organic carbon supply rate (though this was not measured), if the cyanobacterial population (the source of organic carbon) became nutrient limited (or, in the case of high UV treatments, suffered mortality). Thus we speculate that the shift towards het2 and het3 corresponded with a change in organic carbon availability, which favored growth of the putative aerobic anoxygenic photoheterotrophs het2 and het3 over the non-phototrophic het1. This remains to be tested through isolation, physiological characterization, and genome reconstruction of the dominant heterotrophs.

**Lotka-Volterra Model Description**

*Standard Lotka-Volterra model*

We developed a standard Lotka-Volterra model to describe the growth of a bacterial species in an environment with a limited resource. The carrying capacity *K* denotes how many bacteria are supported by the available nutrient concentration.

Because the media is resource-limited and the growth curves indicate growth consistent with a limited resources (e.g. phosphate), we assume that this serves as an appropriate starting point for modeling the interaction of the heterotroph species. We explicitly model each species that undergoes a large change in abundance in the biomass removal (BR) experiments.

The differential equation describing a resource-limited Lotka-Volterra model is shown below, where *h_i_* stands for all the heterotrophs modeled.


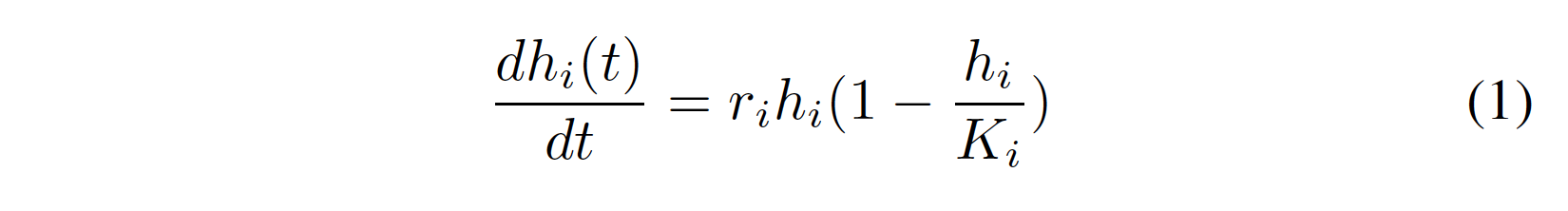


*Competitive Lotka-Voltera model*

In addition to the standard Lotka-Volterra model, one can add competition between species by adding a competition term *c_ij_*. We found that there likely exists a mutual competition between het1 and het2 because we could only reproduce the surge of het2 upon the decline of het1 when we added a competition term for het2 and het1. We can write this extension as follows:


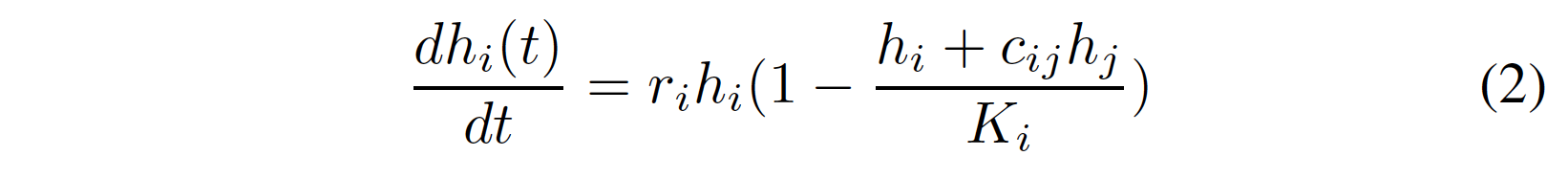


*Coupling resource dependency to the Lotka-Volterra model*

The original LV equation includes a carrying capacity, which can be interpreted as a term that indirectly models the finite nature of the resources needed to sustain a bacterial population. Additionally, the experiments show a potential resource dependence: the het1 species abundance strongly correlates with the amount of free phosphate in the media (Fig. 2). We can therefore add a more direct dependence of het1 growth on a free resource (*P*) in the following equation:


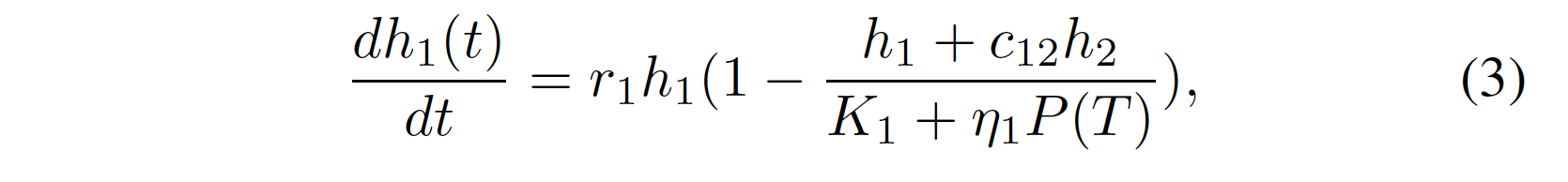


*P(t)* is the time-dependent free resource in the media. Assuming that each species consumes resource *P* from the media, we say that each species consumes the resource when growing, and releases it when it dies (see Equation 4).


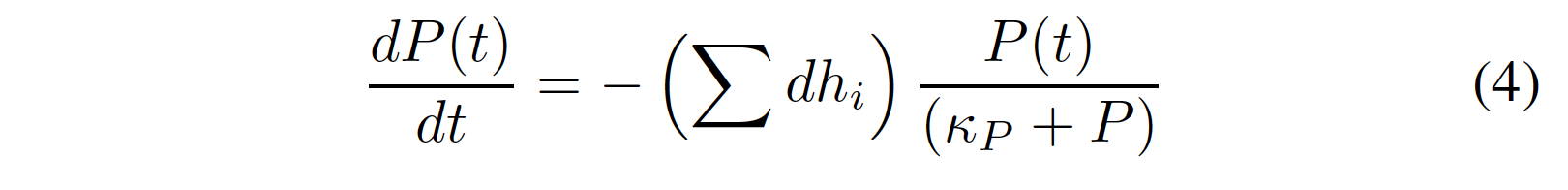


Because het1 does not go extinct entirely when the levels of free resource are low, but is still present at low abundance, we assume it has a low, finite carrying capacity *K_1_*, where the bacteria persist in a dormant metabolic state (see parameter table below).


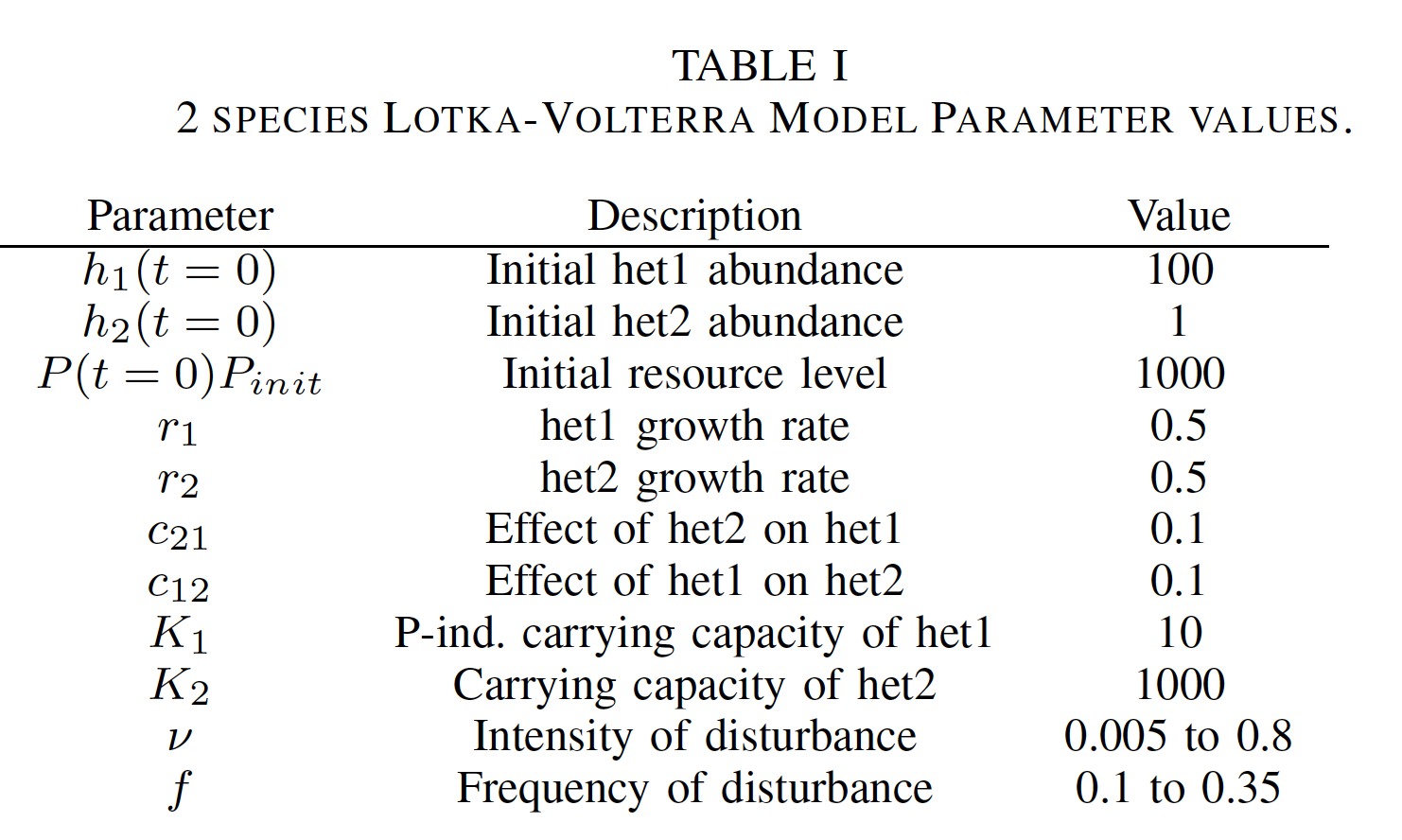


*Biomass Removal Disturbance*

The biomass removal disturbance consists of the removal of 5%, 10%, or 15% of the biomass every 1, 2, or 4 days.

To simulate this, we assign the removal amount *ν* and the frequency a value *f*. We model this with a *δ* function that removes a fraction *ν* with period *t_dist_*.

*Two-species Lotka-Volterra model*


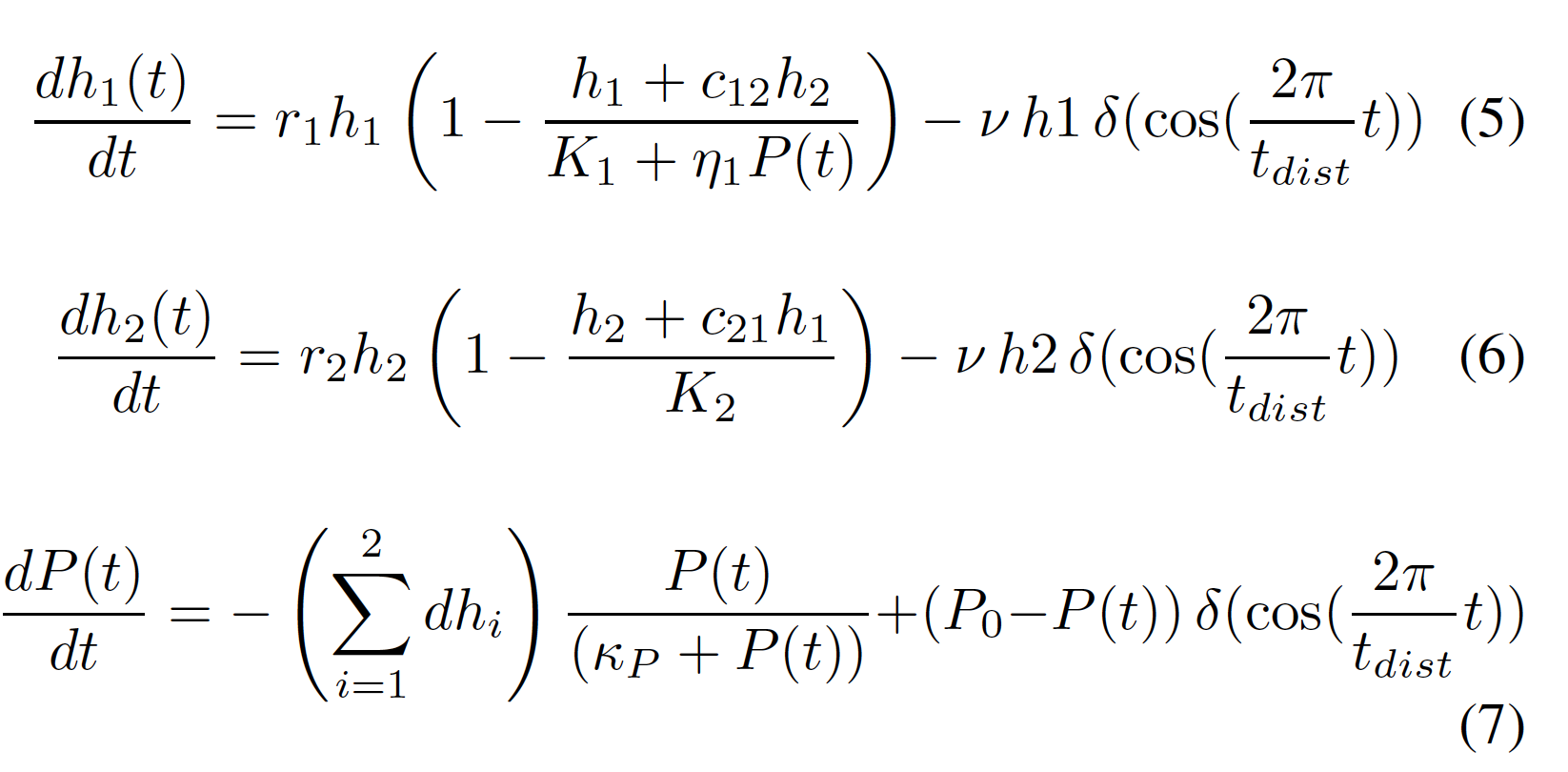


Having explained our approach via the Lotka-Volterra (LV) formulation, we want to understand the dynamics between the resource-dependent switch between het1 and het2.

We can express the ratio between het1 and het2 as *α*, which is large, when het1 is dominant, and small, when het2 is dominant. As Fig. 5 shows, a simple two-species LV model can reconstitute key features of a resource-dependent switch, which results in a humped DDR (Fig. 6A). This model exhibits a simple multiplicative response to disturbance; the diversity depends on the product of intensity and frequency (i.e. the disturbance rate), rather than a more complicated interaction suggested by prior work (7). The parameters for this model are shown in the table above.

**Supplemental References**

1. **Rosenberg E, DeLong EF, Lory S, Stackebrandt E, Thompson F.** 2014. The Prokaryotes: Alphaproteobacteria and Betaproteobacteria. Springer Berlin Heidelberg.

2. **Li X, Koblížek M, Feng F, Li Y, Jian J, Zeng Y.** 2013. Whole-genome sequence of a freshwater aerobic anoxygenic phototroph, Porphyrobacter sp. strain AAP82, isolated from the Huguangyan Maar Lake in southern China. Genome Announc **1:**e00072-00013.

3. **Coil DA, Flanagan JC, Stump A, Alexiev A, Lang JM, Eisen JA.** 2015. Porphyrobacter mercurialis sp. nov., isolated from a stadium seat and emended description of the genus Porphyrobacter. PeerJ **3:**e1400.

4. **Hiraishi A, Kuraishi H, Kawahara K.** 2000. Emendation of the description of Blastomonas natatoria (Sly 1985) Sly and Cahill 1997 as an aerobic photosynthetic bacterium and reclassification of Erythromonas ursincola Yurkov et al. 1997 as Blastomonas ursincola comb. nov. Int J Syst Evol Microbiol **50:**1113-1118.

5. **Yurkov VV, Beatty JT.** 1998. Aerobic anoxygenic phototrophic bacteria. Microbiol Mol Biol Rev **62:**695-724.

6. **Hauruseu D, Koblížek M.** 2012. Influence of light on carbon utilization in aerobic anoxygenic phototrophs. Appl Environ Microbiol **78:**7414-7419.

7. **Miller AD, Roxburgh SH, Shea K.** 2011. How frequency and intensity shape diversity–disturbance relationships. P Natl Acad Sci USA **108:**5643-5648.
