# Supplementary figures and images for: Disturbance Regimes Predictably Alter Diversity in an Ecologically Complex Bacterial System
Source: mBio. 2016 Dec 20;7(6):e01372-16. doi: 10.1128/mBio.01372-16 (PMC5181773; doi:10.1128/mBio.01372-16)

# Biomass Removal

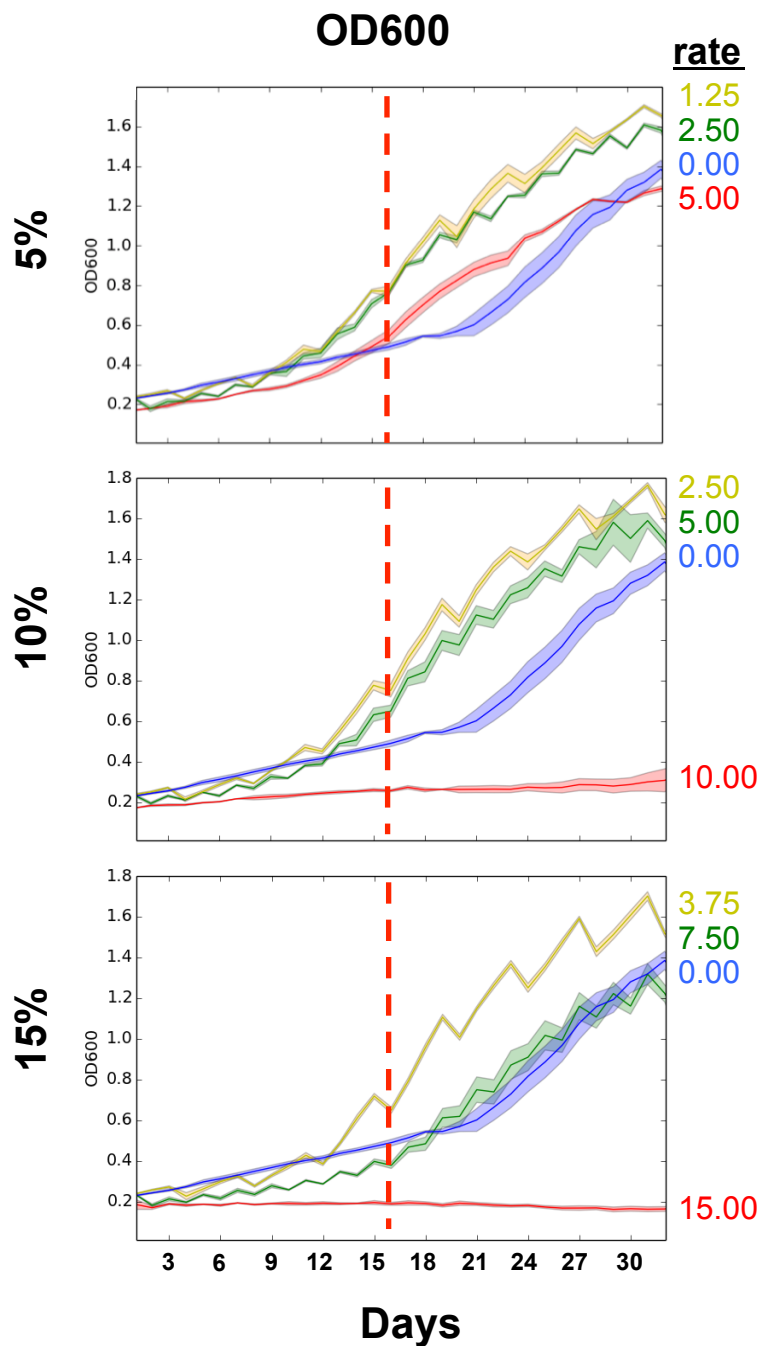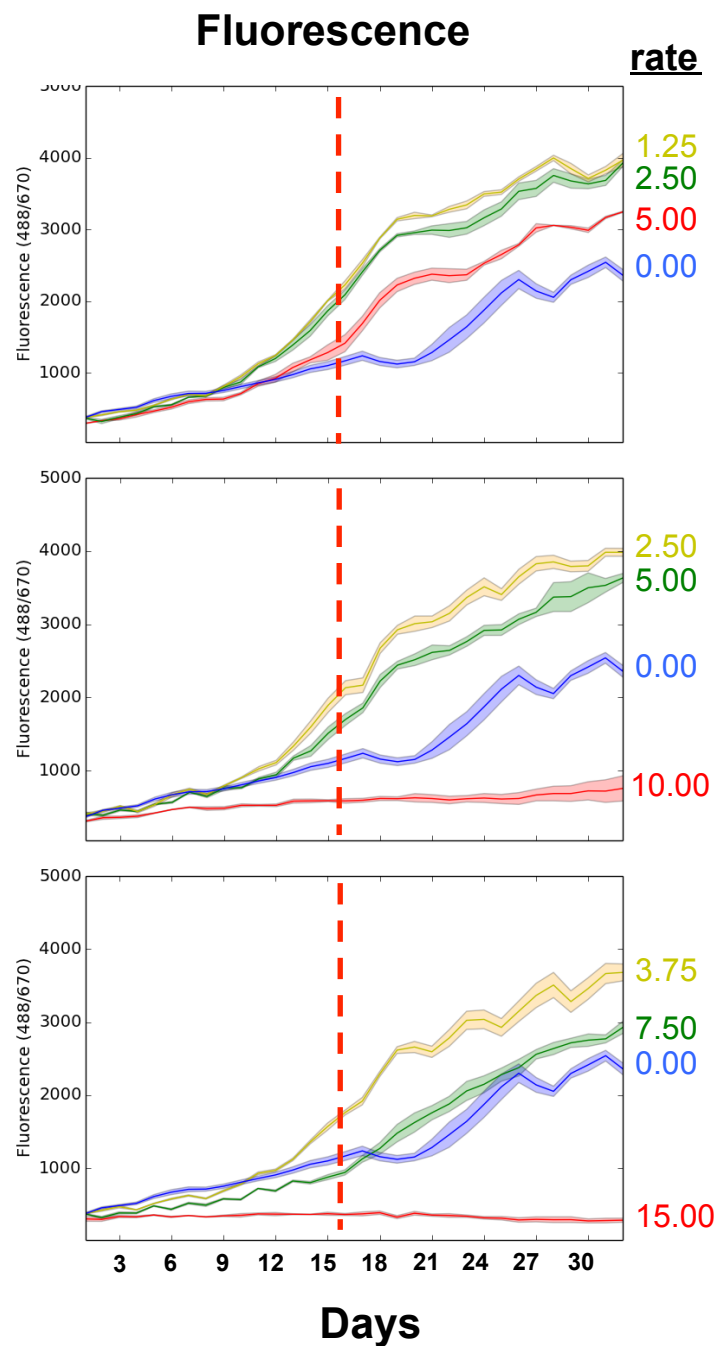

Supplement: Figure S1 — Growth curves for biomass removal treatments (OD600 and fluorescence at 488/670). Disturbances start at day 1 and extend to day 32. Intensity treatments (5, 10, and 15%) are plotted separately, for clarity. Colors indicate disturbance frequency (red, 1 day−1; green, 0.5 day−1; yellow, 0.25 day−1; blue, undisturbed). Disturbance rates are annotated to the right of each curve. Undisturbed controls are plotted in all panels for comparison. The colored area surrounding lines indicates the SD of spectrophotometric measurements across replicates. Vertical dashed lines show sampling day 16. Download [file mbo006163113sf1.pdf]

**A.**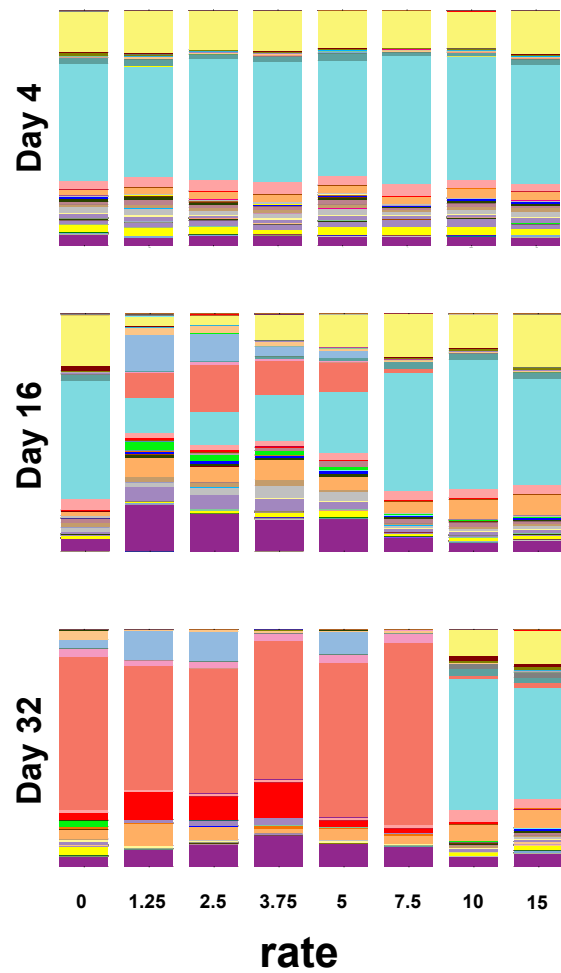**B.**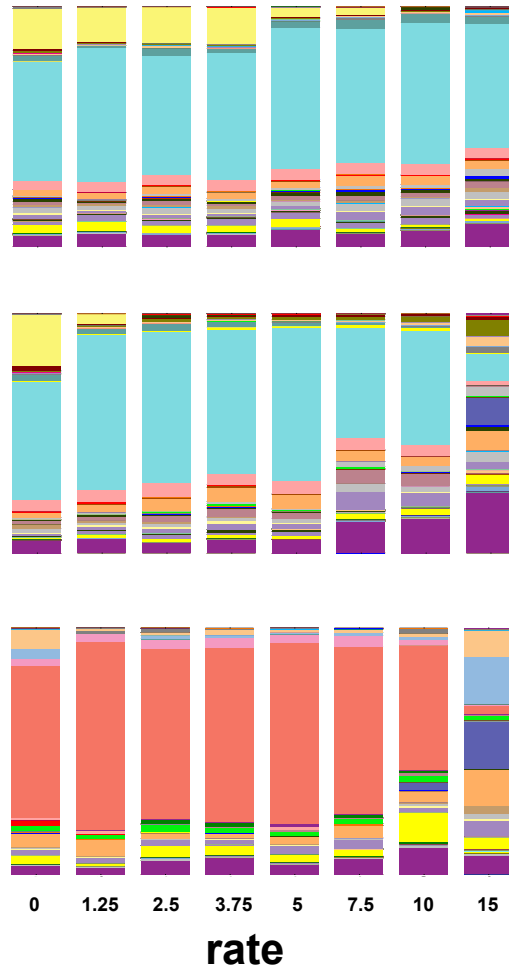**C.**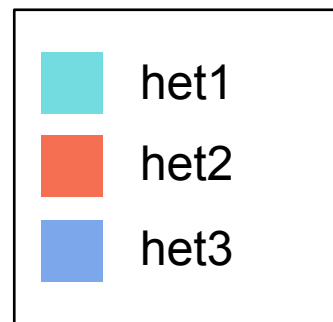**Second UV Experiment**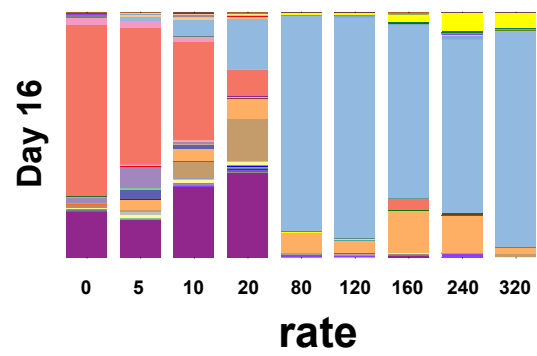

Supplement: Figure S2 — Relative abundances of heterotroph genera for all biomass removal (A) and UV (B) rates on days 4, 16, and 32 and for the second UV experiment (C). The het1, het2, and het3 OTUs comprise >99% of the sequence reads for their respective genera. Download [file mbo006163113sf2.pdf]

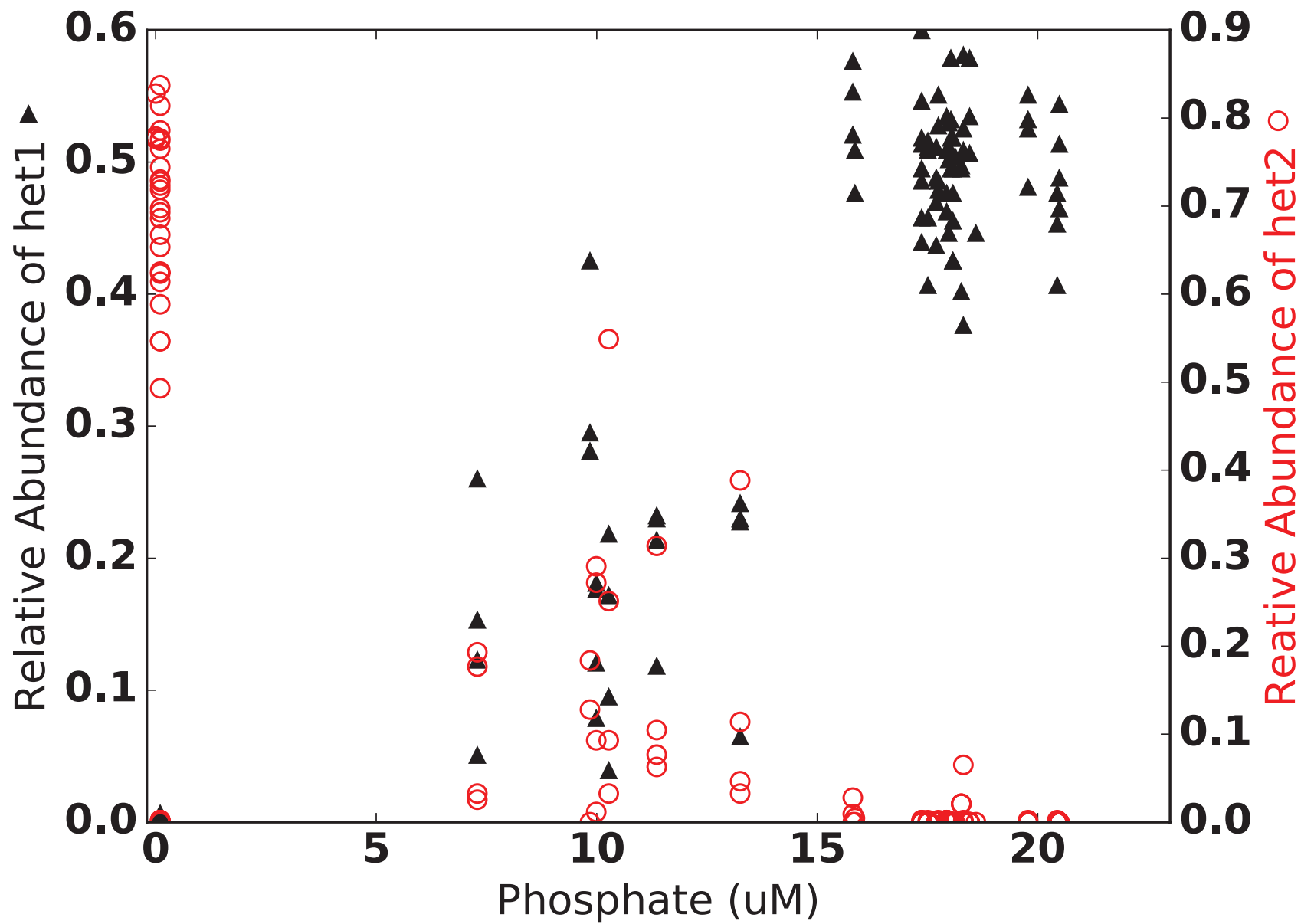

Supplement: Figure S3 — Relative abundances of het1 (black) and het2 (red) across a range of phosphate concentrations. All biomass removal treatment data are included across all time points. Download [file mbo006163113sf3.pdf]

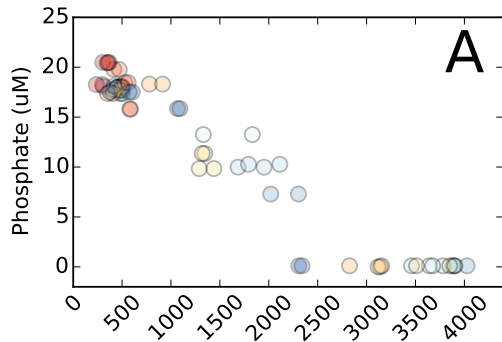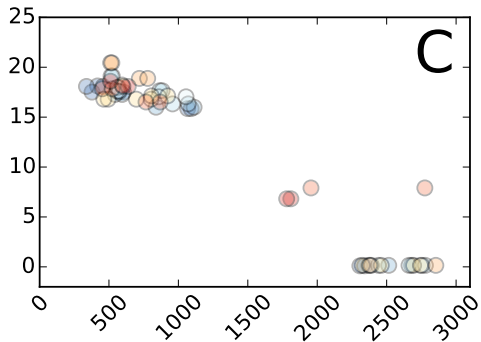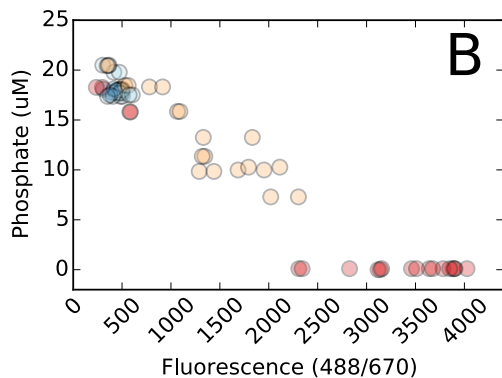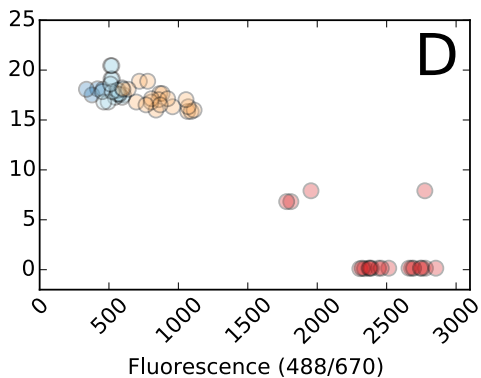

Supplement: Figure S4 — Relationship between phosphate, biomass, and disturbance treatment across time. (A) Phosphate concentration plotted against fluorescence intensity, colored by disturbance rate (biomass removal treatments). (B) Same plot as in panel A, but points are colored by time point. (C) Phosphate concentration plotted against fluorescence intensity, colored by disturbance rate (UV treatments). (D) Same plot as in panel C, but points are colored by time point. Download [file mbo006163113sf4.pdf]

# UV Radiation

5 min.

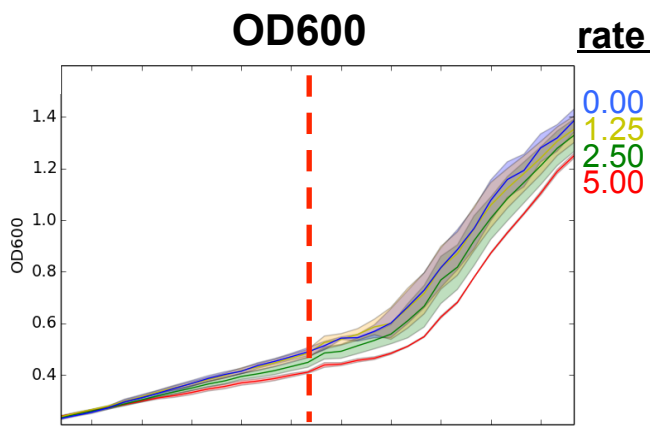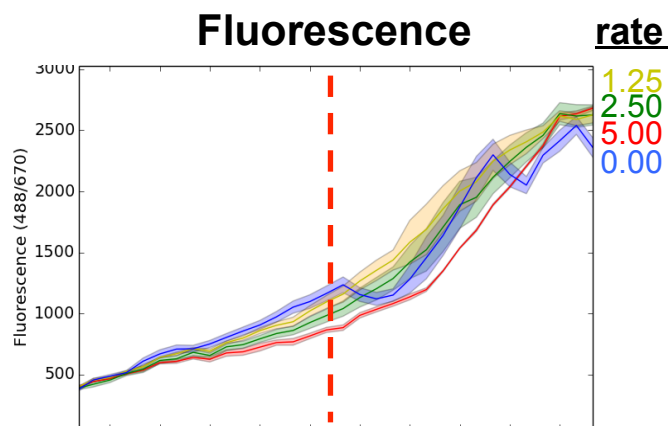

10 min.

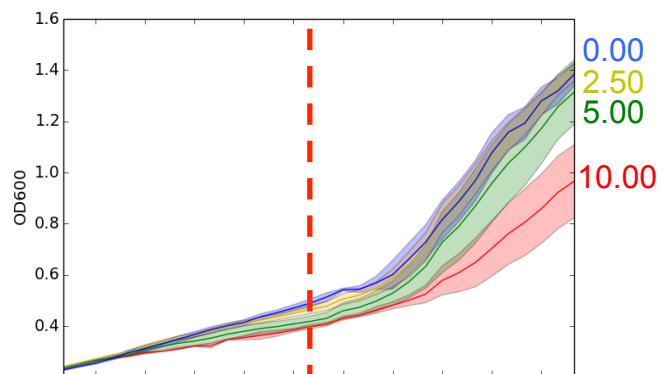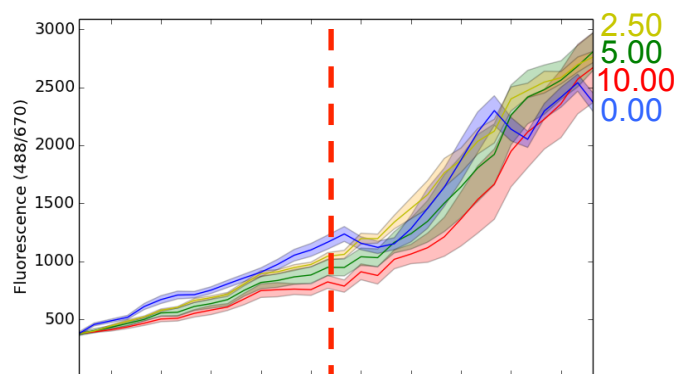

15 min.

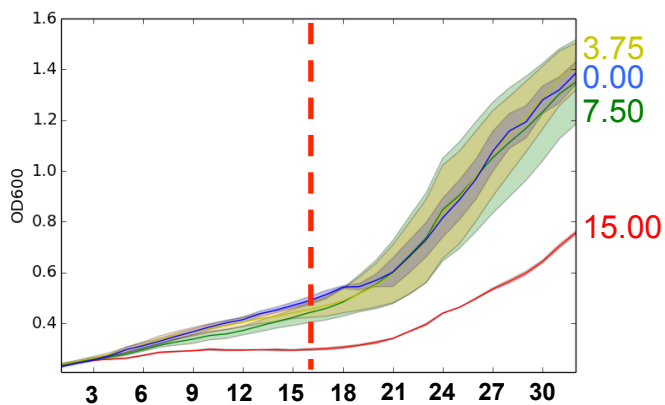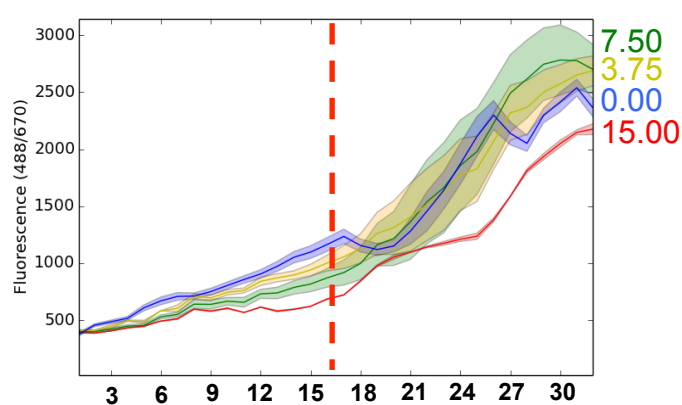

Days

Days

Supplement: Figure S5 — Growth curves for first UV experiment (OD600 and fluorescence at 488/670). Disturbances start at day 1 and extend to day 32. Intensity treatments (5, 10, and 15 min) are plotted separately for clarity. Colors indicate disturbance frequency (red, 1 day−1; green, 0.5 day−1; yellow, 0.25 day−1; blue, undisturbed). Disturbance rates are annotated to the right of each curve. The colored area around lines indicates the SD of spectrophotometric measurements across replicates. Vertical dashed lines show sampling day 16. Download [file mbo006163113sf5.pdf]

20 min. (1X)

OD600

rate

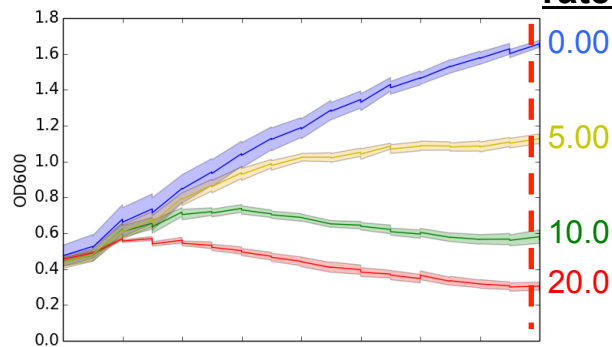

40 min. (8X)

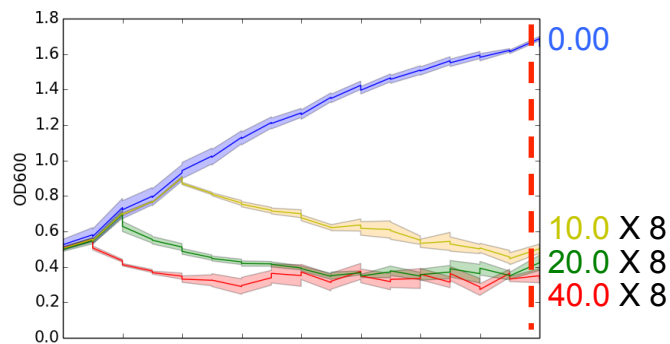

60 min. (8X)

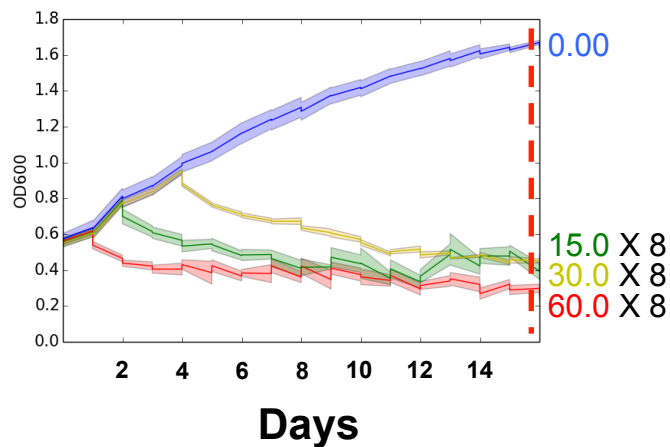

Fluorescence

rate

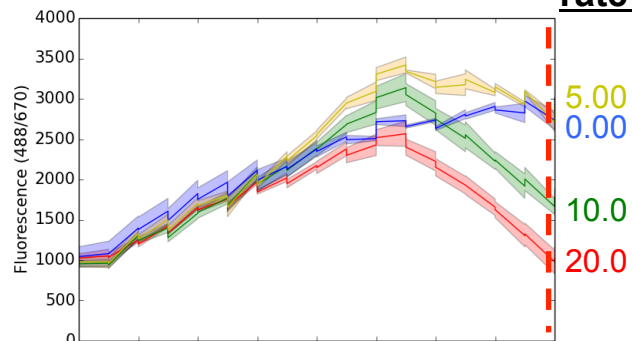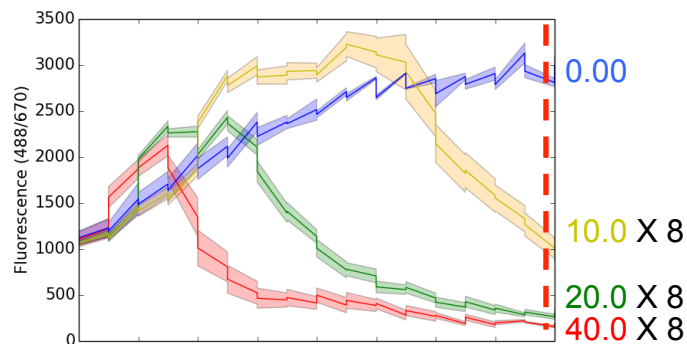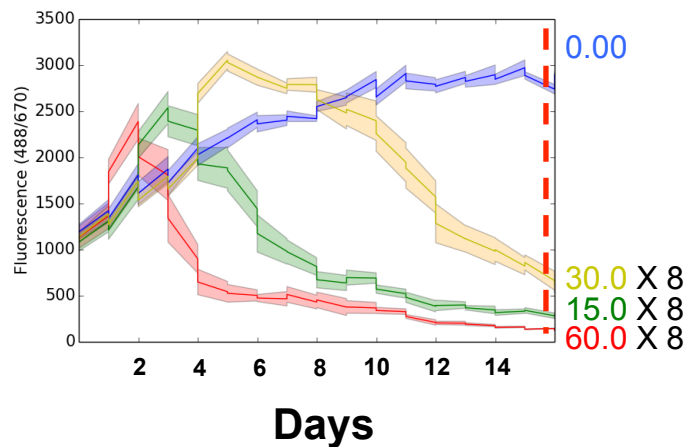

Supplement: Figure S6 — Growth curves for UV treatments from the second experiment (OD600 and fluorescence at 488/670). Disturbances start at day 1 and extend to day 16. Intensity treatments (20, 40, and 60 min) are plotted separately, for clarity. Colors indicate disturbance frequency (red, 1 day−1; green, 0.5 day−1; yellow, 0.25 day−1; blue, undisturbed). Disturbance rates are annotated to the right of each curve. The designations 1× and 8× refer to UV intensity. (Intensity was modulated by changing the proximity of the plates to the UV bulb; radiation intensity decreases as the inverse square of the distance from the source.) The colored area around lines indicates the SD of spectrophotometric measurements across replicates. Vertical dashed lines show sampling day 16. Download [file mbo006163113sf6.pdf]

**A.**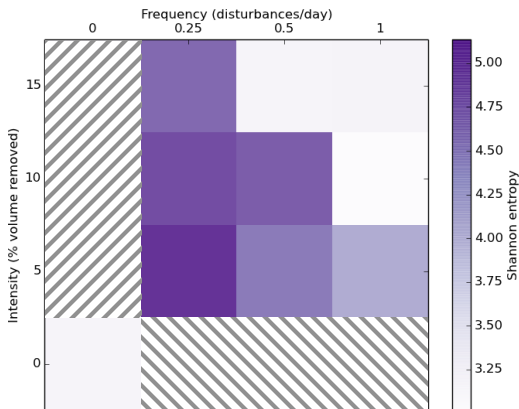**B.**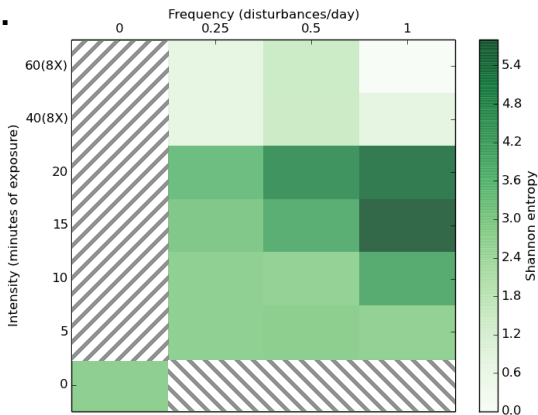

Supplement: Figure S7 — Empirically sampled DDRs for biomass removal and UV treatments, along frequency and intensity axes. Panels A and B show Shannon diversity heat maps for biomass removal and UV exposure treatments, respectively, for day 16 (heterotroph community). Diagonal lines indicate unfeasible combinations. (Without disturbance, there can be no variability in disturbance intensity or frequency.) In panel B, “8×” following the highest intensity treatments refers to a higher applied UV intensity (i.e., the plates were brought closer to the lamp, equivalent to an 8-fold increase in UV flux). Download [file mbo006163113sf7.pdf]

**A.**

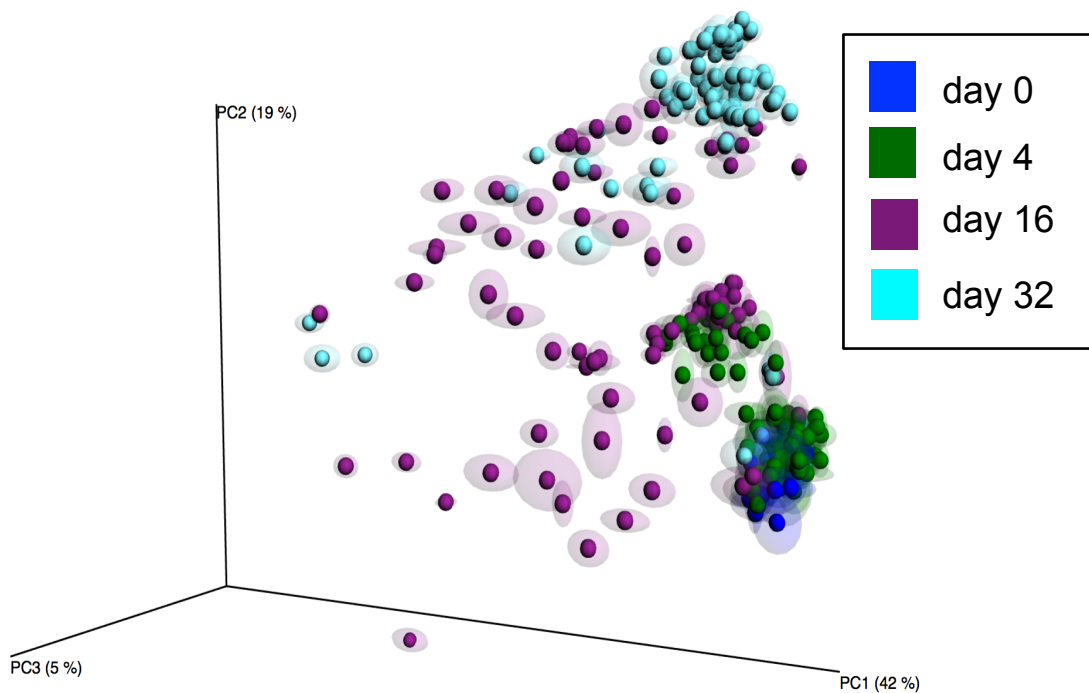

**B.**

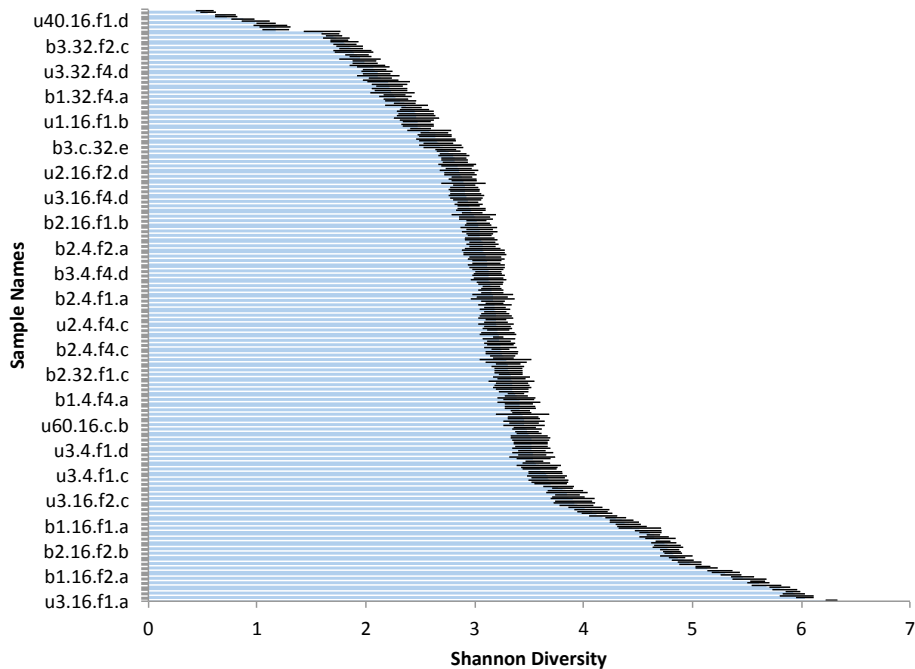

Supplement: Figure S8 — (A) Jackknifed beta diversity analysis (based on weighted UniFrac distances) colored by time point. Translucent bubbles around points indicate the uncertainty (SD) in the principal coordinates of a sample after multiple resampling (with replacement) at 430 sequences (n = 10). Overall, beta diversity patterns are robust to random resampling at 430 sequences per sample, indicating that 430 sequences are enough to accurately describe beta diversity patterns. (B) Jackknifed Shannon diversity analysis for every sample in the data set. Blue bars show mean Shannon diversity, and black lines show the uncertainty (SD) in Shannon diversity after multiple random subsamplings to a depth of 430 sequences per sample (n = 10). Download [file mbo006163113sf8.pdf]
