# Supplementary material for: Disturbance Regimes Predictably Alter Diversity in an Ecologically Complex Bacterial System
Source: mBio. 2016 Dec 20;7(6):e01372-16. doi: 10.1128/mBio.01372-16 (PMC5181773; doi:10.1128/mBio.01372-16)
Supplement: Table S1 — Taxonomic composition of the enrichment stock culture used to initiate disturbance experiments, summarized at the genus level. Relative abundances of 15 dominant heterotrophic genera are shown at two different collection dates, along with the most resolved Greengenes annotation available. The phosphate concentration was high when the sample was collected in August (>10 μM) and low when the sample was collected in November (<1 μM). The genera containing het1, het2, and het3 OTUs are highlighted in bold; the het1, het2, and het3 OTUs each accounted for >99% of the reads in their respective genera. [file mbo006163113st1.pdf]

Table S1. Taxonomic composition of the enrichment stock culture used to initiate disturbance experiments, summarized at the genus level. Relative abundances of 15 dominant heterotrophic genera are shown at two different collection dates, along with the most resolved Greengenes annotation available. The phosphate concentration was high when the sample was collected in August (> 10 μM) and low when the sample was collected in November (< 1 μM). The genera containing het1, het2, and het3 OTUs are highlighted in bold; the het1, het2 and het3 OTUs each accounted for >99% of the reads in their respective genus.

	**Aug. 2013 (high PO_4^{3-})**	**Nov. 2013 (low PO_4^{3-})**
**het1 genus** (*Alphaproteobacteria*, *Sphingomonadales, Sphingomonadaceae*)	**0.127 ± 0.034**	**0.000 ± 0.000**
**het2 genus** (*Alphaproteobacteria*, *Sphingomonadales, Erythrobacteraceae*)	**0.066 ± 0.006**	**0.440 ± 0.015**
Polaromonas (*Betaproteobacteria, Burkholderiales, Comamonadaceae*)	0.119 ± 0.010	0.084 ± 0.016
Flavobacterium (*Bacteroidetes*, *Flavobacteriales*, *Flavobacteraceae*)	0.082 ± 0.018	0.100 ±0.024
Chitinophagaceae genus (*Bacteroidetes*, *Sphingobacteriales*)	0.054 ± 0.020	0.061 ± 0.012
Caulobacteraceae genus (*Alphaproteobacteria*, *Caulobacterales*)	0.033 ± 0.012	0.043 ± 0.023
Sphingomonadales genus (*Alphaproteobacteria*)	0.047 ± 0.012	0.016 ± 0.005
Mycoplana (*Alphaproteobacteria*, *Rhizobiales*, *Brucellaceae*)	0.047 ± 0.011	0.015 ± 0.007
Bradyrhizobiaceae genus (*Alphaproteobacteria*, *Rhizobiales*)	0.010 ± 0.008	0.047 ± 0.010
Rhizobiales genus (*Alphaproteobacteria*)	0.018 ± 0.004	0.023 ± 0.006
Sphingomonadaceae genus (*Alphaproteobacteria*, *Sphingomonadales*)	0.020 ± 0.015	0.015 ± 0.007
Hyphomonadaceae genus (*Alphaproteobacteria*, *Rhodobacterales*)	0.000 ± 0.000	0.029 ± 0.007
**het3 genus** (*Alphaproteobacteria*, *Sphingomonadales, Sphingomonadaceae*)	**0.017 ± 0.010**	**0.005 ± 0.006**
Rhodobacter (*Alphaproteobacteria*, *Rhodobacterales*, *Rhodobacteraceae*)	0.012 ± 0.002	0.010 ± 0.005
Bradyrhizobium (*Alphaproteobacteria*, *Rhizobiales*, *Bradyrhizobiaceae*)	0.006 ± 0.006	0.012 ± 0.001
